# Cell type–specific interpretation of noncoding variants using deep learning–based methods

**DOI:** 10.1093/gigascience/giad015

**Published:** 2023-03-27

**Authors:** Maria Sindeeva, Nikolay Chekanov, Manvel Avetisian, Tatiana I Shashkova, Nikita Baranov, Elian Malkin, Alexander Lapin, Olga Kardymon, Veniamin Fishman

**Affiliations:** AIRI, Moscow, 121170, Russia; AIRI, Moscow, 121170, Russia; AIRI, Moscow, 121170, Russia; AIRI, Moscow, 121170, Russia; AIRI, Moscow, 121170, Russia; AIRI, Moscow, 121170, Russia; AIRI, Moscow, 121170, Russia; AIRI, Moscow, 121170, Russia; AIRI, Moscow, 121170, Russia; Institute of Cytology and Genetics, Novosibirsk, 630099, Russia; Novosibirsk State University, Novosibirsk, 630090, Russia

**Keywords:** machine learning, epigenetics, cell state

## Abstract

Interpretation of noncoding genomic variants is one of the most important challenges in human genetics. Machine learning methods have emerged recently as a powerful tool to solve this problem. State-of-the-art approaches allow prediction of transcriptional and epigenetic effects caused by noncoding mutations. However, these approaches require specific experimental data for training and cannot generalize across cell types where required features were not experimentally measured. We show here that available epigenetic characteristics of human cell types are extremely sparse, limiting those approaches that rely on specific epigenetic input. We propose a new neural network architecture, DeepCT, which can learn complex interconnections of epigenetic features and infer unmeasured data from any available input. Furthermore, we show that DeepCT can learn cell type–specific properties, build biologically meaningful vector representations of cell types, and utilize these representations to generate cell type–specific predictions of the effects of noncoding variations in the human genome.

## Background

During the development of a multicellular organism, a single cell gives rise to a large diversity of cell types. These cell types dramatically differ from each other, although sharing almost identical DNA sequences. For example, the same promoter sequence may drive drastically different expression levels of a downstream gene depending on cell type. These differences in expression stem from 2 sources. First, sequence-specific binding by *trans*-factors, which are expressed in one cell type but not another, controls transcriptional activity. Second, some loci become epigenetically bookmarked at the stage of progenitor cells, making these loci unresponsive to *trans*-factors expressed later in development. See [1] for a detailed review.

Developmental history and the presence of *trans*-factor together define the *cell state*, explaining differences in epigenetic properties and transcription levels of the same sequence. Cells sharing the same or close states represent a cell type. Due to differences in cell state, genomic variations may have different, cell state–specific transcriptional or epigenetic effects. This makes interpretation of genomic variations, including clinical assessments of noncoding mutations, challenging.

Importantly, cell states could be learned from the genome-wide expression pattern [2, 3] or epigenetic marks [4], allowing clustering of similar cell types based on their genomic properties. Moreover, many epigenetic marks are interdependent [5], making it possible to use only a subset of measured epigenetic characteristics to infer unmeasured properties and define cell state. Due to the complex nature of dependencies between epigenetic marks, machine learning approaches recently gained attention as attractive tools to solve this problem [6]; however, the existing solutions [7] focus on a specific combination of input and target epigenetic properties, lacking generalizability to infer all epigenetic properties from any available input.

Although the cell state is essential to explain differences between cell types, it could not explain differences in the expression levels or chromatin packaging associated with different sequences within the same cell type. To explain these differences, one should consider the diversity of DNA sequences. There are several computational methods allowing the prediction of specific epigenetic properties [8,9], chromatin packaging [10], or expression levels [9] based on the DNA sequences. Among these, the prediction of gene expression changes caused by sequence variations is especially interesting, because such predictions enable clinical interpretation of noncoding variants in the human genome [9]. However, none of the existing predictive approaches allows generalization of the predictions across cell types, limiting the results to a subset of cell types with known expression patterns.

Here, we introduce the concept of cell state learning, which allows us to build latent vector representations of cell types based on available epigenetic input. We show that sequence specificity could be combined with cell type specificity using modern machine learning architectures. We show the feasibility of the developed models for the cell type–specific prediction of epigenetic properties. Importantly, we showed that these predictions allow inferring functional significance of effects caused by noncoding variants in the human genome.

## Results

### Artificial intelligence model allows learning meaningful computer representation of cell types using arbitrary epigenetic data

**Figure 1:**
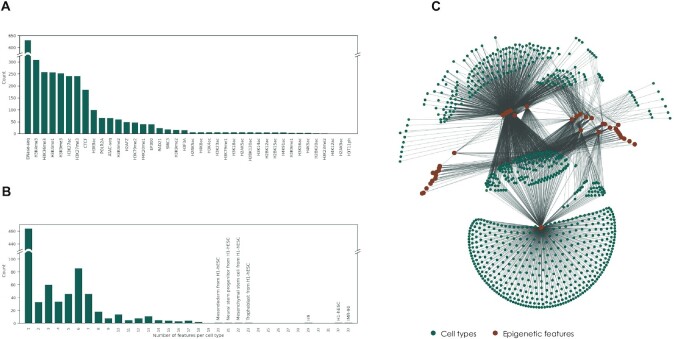
Overview of ENCODE epigenetic datasets characterizing human cell types. (A) Number of cell type–specific epigenetic profiles shown for each epigenetic feature. (B) Histogram showing a number of epigenetic features measured per cell type. (C) Graph representation of measured epigenetic data. Each node presents cell type (green nodes) or epigenetic features (brown nodes), and each edge shows measured cell type–feature track.

We started our investigation by exploring the latest update of the ENCODE dataset [11]. Focusing on human data, we collected information about 858 cell types characterized by 40 epigenetic features, 3,026 tracks in total (Fig. 1). Among these epigenetic features, measurements of DNAse accessibility are the most abundant (available for 631 cell types), and the less-studied marks are H2AK9ac, H4K12ac, and H3T11ph, which are measured only in 1 cell type (Fig. 1A). The most characterized cell type includes IMR-90 fibroblasts, with 33 of 40 epigenetic tracks available; at the same time, for 464 cell types, only 1 epigenetic mark was measured (Fig. 1B). Overall, we found that available information about epigenetic characteristics of human cells is sparse, with only 8.8% of epigenetic tracks present in the ENCODE collection.

We made an assumption that if 2 epigenetic features were measured in 1 cell type, it is possible to learn how the signal of 1 feature depends on the signal of another feature. We also assumed that dependencies between epigenetic features are largely shared between cell types. This assumption might not hold if there are cell type–specific epigenetic mechanisms. However, measured data should allow clustering of epigenetically similar cell types, which probably share mechanisms of epigenetic regulation. Within these clusters, we can utilize dependence between signals of 2 features learned in 1 cell type to predict the signal of an unmeasured feature in another cell type.

To explore how often unmeasured features can be inferred from measured data, we visualized and analyzed cell types and features as a graph where edges represent feature measurements (Fig. 1C). We found that this graph is connected; therefore, under the aforementioned assumptions (i.e., if there are learnable correlations between all epigenetic features), any unmeasured feature can be inferred from the available data. Thus, we aimed to develop a computational approach capable of clustering epigenetically similar cell types and infer unmeasured epigenetic features based on available sparse datasets. Toward this aim, we decided to employ state-of-the-art machine learning methods.

**Figure 2:**
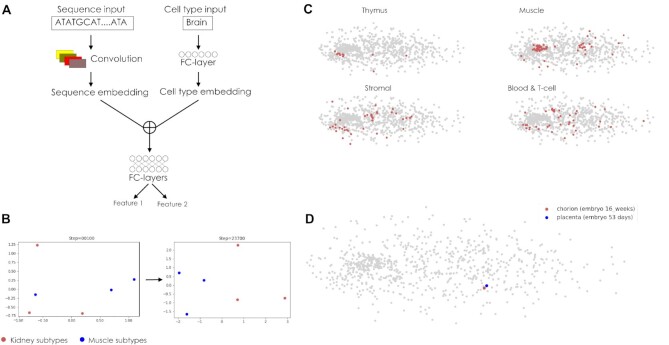
DeepCT models learn computer representations of cell type. (A) An overview of the DeepCT model architecture. Details are provided in methods and Supplementary Note 1. (B) Projection of embeddings for 3 kidney and 3 muscle cell types from 32-dimensional latent space of the model into a 2-dimensional Principal Component Analysis(2D PCA) axis. The figure shows how cell states, initialized randomly, are clustered according to cell identity after ∼23,000 model training steps. (C) Example cell state embedding projections from 32-dimensional latent space of the model into a 2-dimensional PCA axis. Background points show distribution of embeddings for all cell types, whereas colored dots correspond to the specific cell types associated with specific tissue: thymus, muscle, stromal, or blood. (D) DeepCT performs clustering of embryonic and adult heart tissues, although there is no common epigenetic track measured for both samples.

We have designed a new neural network architecture, DeepCT, capturing both sequence- and cell type–specific variations of the epigenetic data (Fig 2A). The model accepts DNA sequence and cell type label as its inputs. The DNA tail processes the nucleotide sequence and builds its computational representation using a deep convolutional neural network (CNN). The cell state tail accepts a 1-hot encoded vector of the input cell type and returns the embedding vector, which represents this cell type in the model's latent space. The sequence and cell state representations are concatenated and fed to the network's head, which outputs sequence- and cell state–specific predictions about the presence of each epigenetic mark.

The idea of cell state learning described above could be implemented using different neural network architectures. We demonstrated this by designing several implementations of the DeepCT model (Methods). The qualitative implementation (DeepCT) aims to solve classification problems (i.e., the presence or absence of a peak at a specific locus in a specific cell type). The quantitative representation (qDeepCT) solves the regression problems, predicting the ChIP-seq, ATAC-seq, DNAse I hypersensitivity, or CAGE data quantitatively. The quantitative implementations use CNN for sequence processing (CNN-qDeepCT and trans-qDeepCT). Following, we provide results for the CNN-qDeepCT model, because it shows better performance on our data.

We expected that during training, the network will fit its parameters so that similar sequences obtain similar embeddings and, likewise, similar cell types will be clustered within the model's latent space. Indeed, a toy example of 6 cell types characterized by a single epigenetic feature shows how clustering of similar (sub)types occurs during neural network training (Fig. 2B; Supplementary Video S1).

Applying DeepCT to the full dataset (excluding data obtained after treating cells with specific compounds) of 2,629 high-quality tracks, we observed that cell type clustering was often concordant with biological expectations (Fig. 2C, D; Supplementary Fig. S1). For example, we show colocalization of muscle cells, as well as colocalization of thymus cells (Fig. 2C, Supplementary Fig. S3). However, other cell types, such as blood or stromal cells, do not form well-defined clusters (Fig. 2C). This might be due to either higher heterogeneity of these tissues or the peculiar properties of the epigenetic data available for these cell types.

To quantify the performance of clustering, we measured how far the cell types of the same tissue or organ appeared to be located within the model's latent space (Supplementary Fig. S1). This analysis confirmed that average cosine similarity for embeddings representing cell types from the same tissue was significantly higher than for embeddings of randomly selected cell types (0.44 vs. 0.3917 ± 0.0029 at random, *P* < 1.3e-62). This result shows that the DeepCT model captures the biological properties of cells during training.

We note that DeepCT learned the cell representation based on different collections of epigenetic marks available for each cell type. For example, we observed clustering of chorion and placenta samples collected at different developmental time points (Fig. 2D), although the set of epigenetic tracks measured in these tissues does not contain common tracks (H3K27me3, H3K4me3, H3K27ac, H3K4me1, and H3K36me3 were measured for the chorion sample; only DNAse I hypersensitivity was measured for placenta). This is possible because all available epigenetic data are fed to 1 model, sharing the latent space and thus allowing multitask training. In this example, we may speculate that DNAse I hypersensitivity and H3K4me1/3 are highly correlative, which was learned from those cell types where both features are available. This correlation can be utilized to infer H3K4me1/3 data in placenta and cluster tissues. We searched for all cases when 2 samples attributed to the same tissue/cell type category are the closest neighbors according to the distances computed in the embedding space but do not share epigenetic tracks. In total, we observed 14 cases of such clustering based on indirect information, which was almost 2 times more than expected at random (7.72 ± 2.97).

Thus, using the developed architecture allows the cell type embeddings to be fitted using any subset of the epigenetic datasets. These embeddings can be next used to infer unmeasured features.

### DeepCT model generalizes across cell types, sequences, and epigenetic tracks

**Figure 3:**
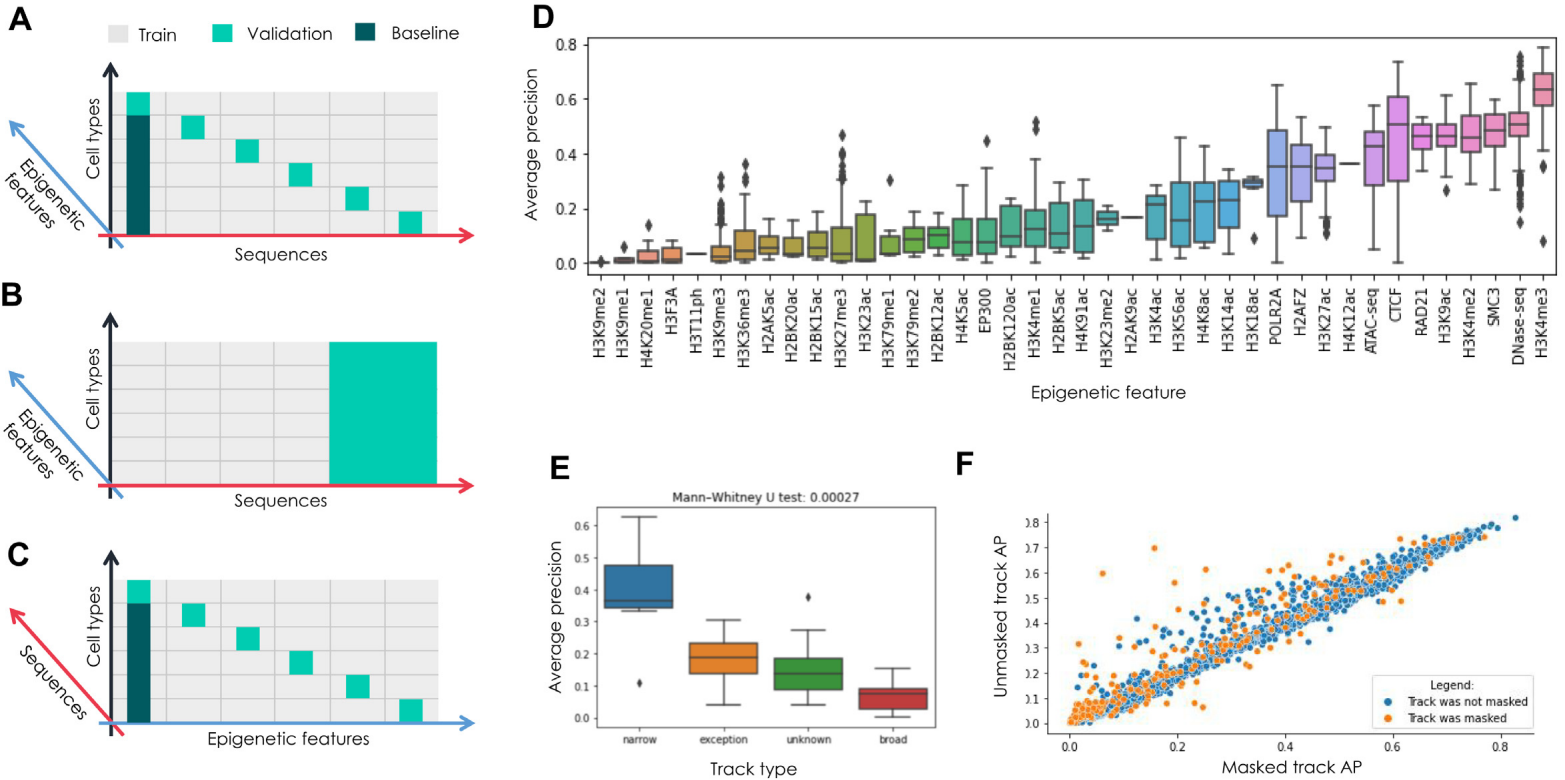
Benchmarking DeepCT architecture. (A–C) Different schemes of DeepCT benchmarks. We show 3 dimensions of the target epigenetic signal values predicted by the neural network: sequence dimension (representing diversity of genomic loci), cell type dimension, and epigenetic feature dimension. Each target value is defined by a combination of genomic loci, epigenetic feature, and cell type. Colors show which targets were excluded from training data. Note the axis swap in panel C: in A and B, we use at least 50% of examples for any cell type × feature combinations for model training, whereas for C, we completely removed data for some of the cell type × feature combinations from the training dataset. (D) Distributions of average precision (AP) values obtained for different cell types. AP was measured for unseen sequences. (E) Distributions of AP values shown for different feature classes. Narrow features display significantly higher AP scores than broad features (*U*-test = 0.00027). (F) Scatterplot showing how AP changes when excluding epigenetic track from training. The x-axis shows AP measured for a track included in training (i.e., track's data were included for training sequences set). The y-axis shows AP measured for the same track, but in this case, no track's data were included during training, requiring the model to infer the track values from the cell state embedding. In both cases, AP was measured for the same set of unseen sequences.

To benchmark the developed DeepCT models, we considered 3 challenges (Fig. 3A–C). For the first, cell type specificity challenge, we excluded 10% of epigenetic data for each locus and evaluated the model on these excluded targets (Fig. 3A; see also details in Methods). Note that in this setup, for each locus, the training data include targets measured for some, but not all, cell types. As a baseline in this challenge, we used the average target value for a given locus across cell types included in training. Biologically, this baseline can be interpreted as an average cell type's epigenetic profile. We note that this baseline has performance metrics substantially higher than expected in random (baseline mean Average Precision(mAP) = 0.446), because differences between sequences explain substantially more variance in epigenetic data than differences between cell types.

We compared performance of the DeepCT models with baseline metrics and found that the model outperforms the baseline, with model mAP of 0.4753 ± 0.0015 (mean over models for 3 different data splits and random seeds) vs. baseline mAP of 0.446. Additionally, for each trained model, we ran a Wilcoxon median test on per-track differences of model and baseline scores and found that model scores were significantly higher than baseline scores (*P* < 2.1e-14). This shows that cell state embeddings are meaningful and that the model predicts cell type–specific differences in epigenetic features using learned embeddings.

Next, we considered the sequence specificity benchmark. In this test, we defined a subset of validation loci and completely excluded any information about these loci from the training dataset. This is a more complicated challenge compared to the previous one when, for each sequence, a subset of target values was available during training. Expectedly, the model's performance was lower in this case (mean AP ∼0.34, mean *r*^2^ ∼0.16). Of note, using previously published DeepSEA architecture showed comparable scores (mean AP ∼0.32), although DeepCT gains ability to infer unmeasured features.

We found that performance of DeepCT significantly varies among features (Fig. 3D). For example, H3K9 and H4K20 monomethylation have almost zero average precision; on the other hand, H3K4me3 mark shows a high AP score (mean across cell types = 0.62), and for best cell types, we achieved AP ∼0.8 on unseen sequences. The predictive power was also high for DNAase I hypersensitivity, CTCF, Cohesin subunits, and some other epigenetic datasets.

To understand the observed difference between features, we classified them according to the ENCODE consortium guidelines to “narrow,” “broad,” and “exception” categories (features that were not attributed to one of these categories were grouped as “unknown”). Features of the first category (“narrow”) show narrow chromatin occupancy signals contained within short (100–200 bp) fragments of genome, whereas chromatin proteins attributed to the “broad” category include continuous histone marks occupying up to several hundred kilobases. We found that narrow features show significantly (Mann–Whitney *U*-test *P* = 0.00027) higher mean average precision values than features of the broad peak's class (Fig. 3E). This difference is likely due to the relatively short input sequence processed by DeepCT, which does not contain all sequence determinants necessary for broad marks inference.

Finally, the most complicated challenge is the prediction of previously unmeasured epigenetic tracks. To benchmark our model, we excluded a subset of tracks from the training dataset. We selected these tracks so that each cell type was characterized by at least 1 epigenetic track, allowing us to learn an embedding for this cell type. In addition, we ensured that each excluded track can be inferred by learning dependencies between epigenetic features from the training data (Methods).

Expectedly, the quality of the predictions was lower for unseen tracks (Fig. 3F). However, the drop of predictive power was relatively small, only 1.6% of the AP score on average. This indicates that DeepCT architecture can be used to infer previously unmeasured data, serving as an important instrument for interpretation of noncoding variants in the human genome.

In order to demonstrate that our model does indeed utilize the contextual information beyond the 200-bp target interval, we have created input saliency maps for 1,000 samples via guided gradient backpropagation (presented in Supplementary Fig. S4). Greater input gradient value implies that changes in this input will have greater impact on the model output, and thus this input has greater contribution to the model prediction. We find that our model does in fact utilize the context and not just the target window for prediction; however, the expected overall pattern is “the further from the target window, the less importance for prediction” (Supplementary Fig. S5).

### Using the DeepCT models for interpretation of clinically significant genomic variants

To benchmark the clinical relevance of the obtained predictions, we employed the collection of genomic variants previously associated with autism spectrum disorder [12,13]. Although epigenetic impact was recently measured for a large collection of noncoding variants [14], for the vast majority of variants identified by [12] and [13], epigenetic effects were not known. To gain this information, we predicted 40 epigenetic characteristics for each single-nucleotide variant (SNV) in the dataset. Comparing predictions obtained for reference and alternative alleles, we scored the effects of SNVs identified in children with autism spectrum disorder and their unaffected siblings.

**Figure 4:**
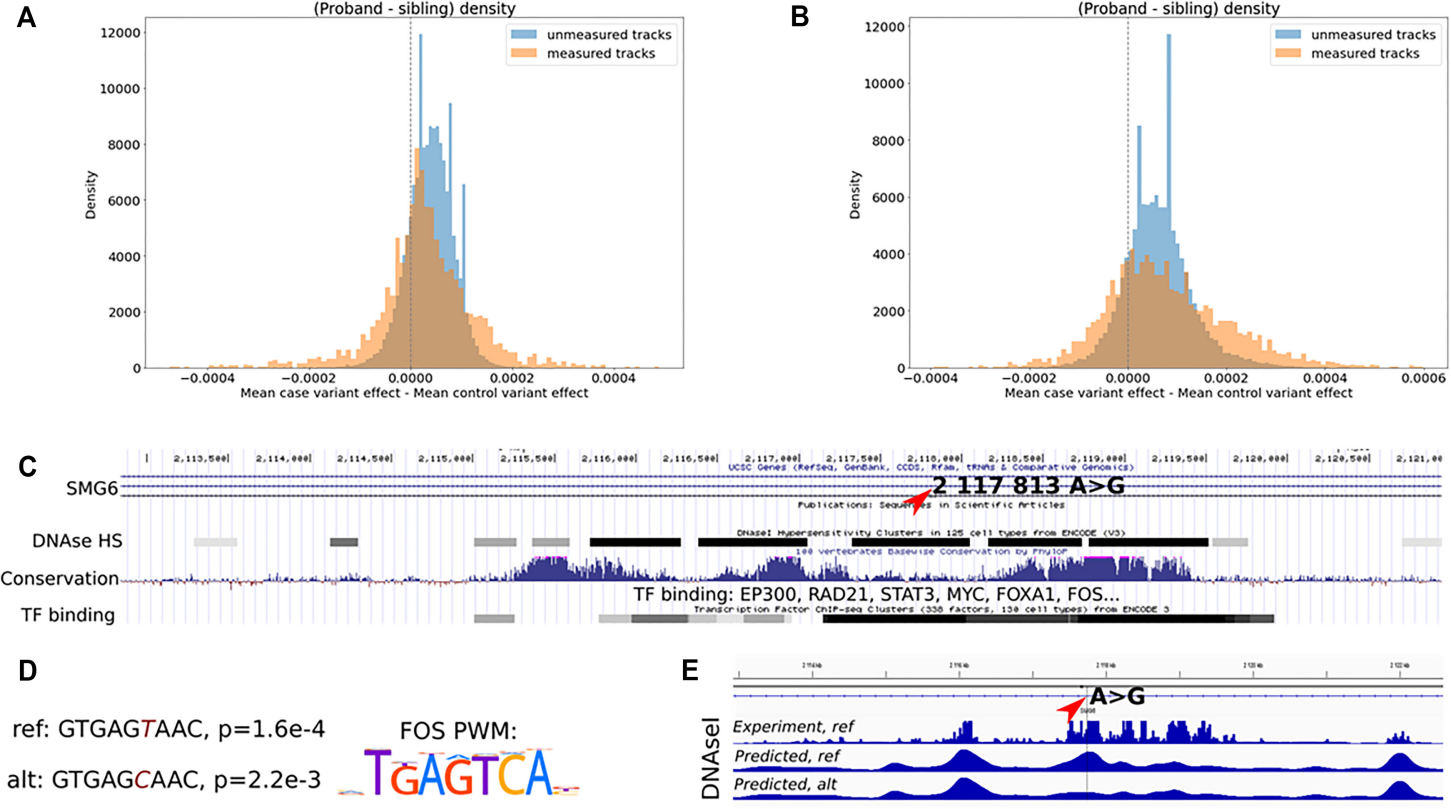
DeepCT predicts effects of SNVs associated with autism spectrum disorders. (A, B) Distribution of mean difference of effect size for the affected child's and unaffected sibling's SNVs. Data are the average across SNVs, that is, each sample in the distribution corresponds to 1 of 31,760 epigenetic tracks (794 cell types × 40 epigenetic features) predicted by DeepCT. Datasets in which experimental measurement for the reference allele was available during the model's training are highlighted as “measured tracks.” Effects of SNVs from [12] are shown in A and from [13] are shown in B. (C) Genomic regions within the SMG6 gene intron containing the NC_000017.10:g.2117813A>G variant (depicted by arrow). (D) FOS motif *P* values for reference and alternative alleles computed based on FOS position weights matrix (PWM). Note that we show the reverse complement of the sequence-matching PWM. (E) DNAse I hypersensitivity tracks for the M059J cell line. Position of NC_000017.10:g.2117813A>G variant depicted by arrow.

We found that effects of variants identified in genomes of children with autism spectrum disorder were on average larger than effects of variants carried by their unaffected siblings (Fig. 4A, B). This difference was observed both for epigenetic tracks where reference alleles were experimentally characterized and for epigenetic tracks that were never measured before.

We focused on specific SNVs with high effect sizes. These SNVs often fall into DNAse I hypersensitive regions with multiple binding sites for key developmental regulators. For example, we identified an SNV within the intron of the *SMG6* gene (NC_000017.10:g.2117813A>G), approximately 50 kbp from its transcription start site (Fig. 4C). Previously, SNVs and copy-number variations affecting these genes were associated with autism spectrum disorders [15–17]. Consistently, DeepCT predictions show the highest impact for the M059J line, which originates from glial cells.

Although there were other SNVs identified within the *SMG6* gene, only this SNV was scored high by DeepCT, predicting almost a 10-fold change of DNAse I accessibility. In accord with the high impact predicted by DeepCT, this SNV falls within an evolutionary conserved genomic locus, demarcated by H3K27 acetylation marks and containing multiple transcription factor binding sites (Fig. 4C). Analysis of transcription factor motifs based on the HOCOMOCO motif search tool [18] shows that the SNV significantly decreases the probability of FOS transcription factor binding in this region (Fig. 4D). Accordingly, DeepCT predicts a substantial decrease of DNAse I sensitivity for an alternative allele (Fig. 4E). These observations can explain how this SNV leads to dysregulation of *SMG6* gene expression and contributes to the development of the autism spectrum disorder.

### DeepCT discriminates causative variants in the GTEx-eQTL dataset

We conducted additional verification of the DeepCT model using GTEx data v7 [19]. Only uniquely matching tissues from the GTEx dataset to cell type tissues were used in the analysis (Supplementary Table S1). For each of such 22 tissues in the GTEx data, we selected top 50 significant variant–gene associations based on permutations [19]. We are only interested in the top-associated variants across loci because our model should score only causal variants as functional. We combined selected variants and got the list of 584 variants, including about 120 significant and 450 nonsignificant variants for each tissue. Next, we used the quantitative DeepCT model to predict functional significance of the selected variants (see Methods). Finally, we applied a *t*-test for DeepCT scores between significant and nonsignificant variants for each tissue and selected features.

**Figure 5:**
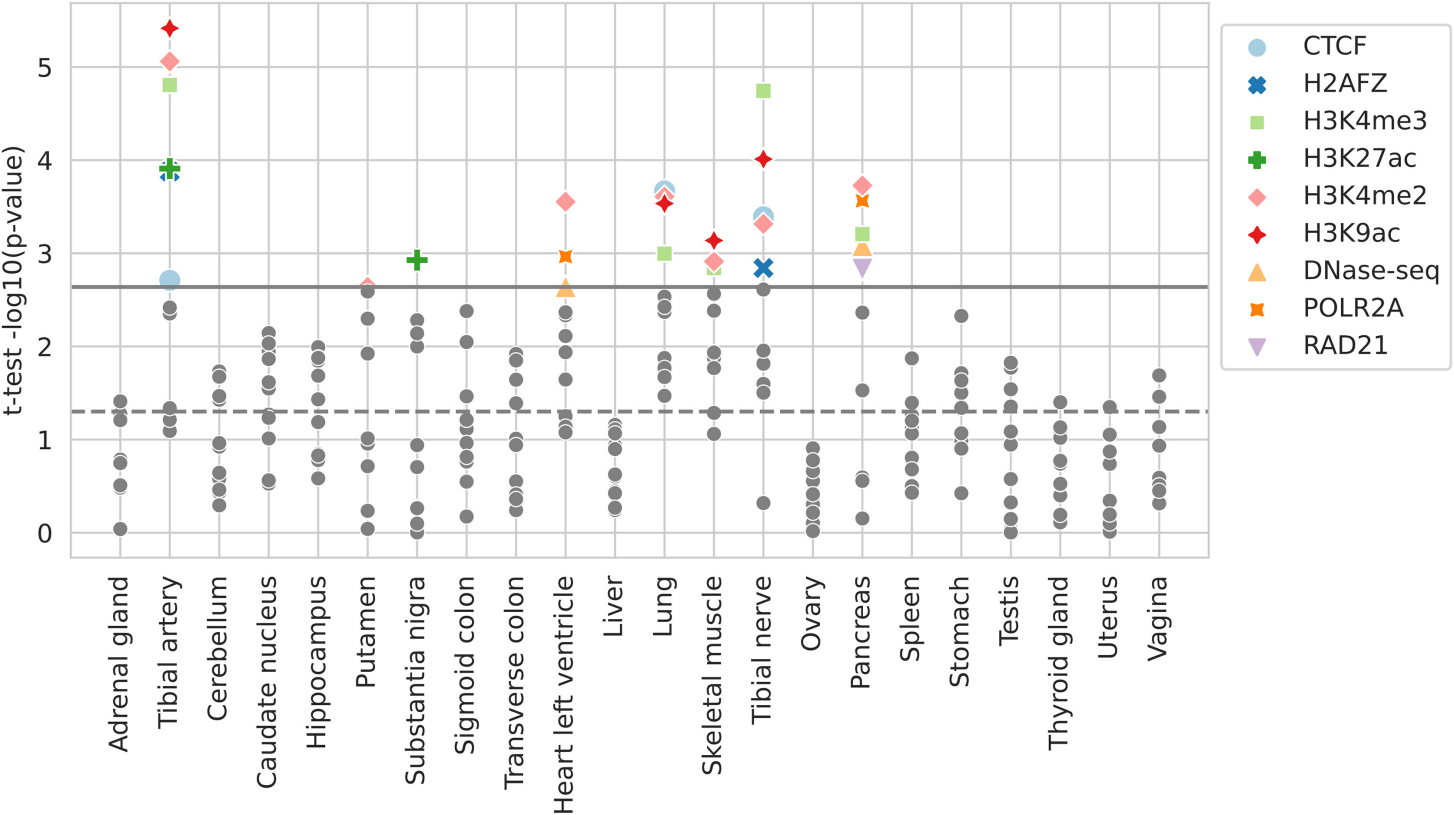
The *t*-test results for comparison of DeepCT scores for significant and nonsignificant variants for each tissue and selected features. Significant results are colored and marked by features, shown in the legend. Nonsignificant results are shown as gray dots. Solid gray line corresponds to the *P* value thresholds (*P* = 0.0023); dot line corresponds to nominal *P* value (*P* = 0.05).

Analysis shows significant results for tibial artery, substantia nigra, heart left ventricle, lung, skeletal muscle, tibial nerve, pancreas, and putamen tissues at least for 1 feature (*t*-test *P* < 0.0023) (Supplementary Table S2, Fig. 5).

The most frequently significant results were observed for H3K4me3, H3K4me2, and H3K9ac, while *t*-test results for SMC3 and ATAC-seq for all tissues were insignificant. Note that model performance for ATAC-seq features has a high dispersion across different cell types (Fig. 3D), which could explain the absence of significant results after averaging by cell types. In addition, H3K4 di- and trimethylation is a hallmark of active promoters, and H3K9ac is essential for pol II pause release [20], which may explain why DeepCT functional scores derived from these features show the highest concordance with expression data. Overall, *t*-test demonstrated concordance results for 8 of 22 GTEx tissues.

There are several reasons why not all significant variants in GTEx summary statistics have a high score in DeepCT: (i) our model provides scores for epigenetic features, which may not always directly influence gene expression; (ii) our model gives a high score only for causative variants, which could be absent in summary statistics (i.e., when only the proxy variant is present); and (iii) finally, the accuracy of prediction may not be high enough to catch the significant effects, for example, if we do not have enough samples/cell types representing specific GTEx tissues.

## Discussion

We challenged one of the most important questions in modern genomics: interpretation of noncoding genomic variants. We show how this question can be answered using state-of-the-art machine learning methods. For this aim, we decomposed epigenetic profiles into 2 components: one representing cell type–specific mechanisms (cell states) and another representing diversity of genomic sequences. Speaking in biological terms, we can imagine that 2 heads of the DeepCT network are responsible for learning mechanisms of *cis-* and a *trans-*regulation, whereas the tail of DeepCT learns interplay between these 2 factors and their epigenetic manifestations.

Although the possibilities opened by DeepCT-based neural network architectures seem promising, we would like to discuss limitations of our approach and directions toward improvement of DeepCT-based architectures.

First, we cannot guarantee generalizability of the model across all human cell types. In fact, we expect that very specific epigenetic profiles, observed, for example, in germ cells, zygotes [21], or some adult cell types, such as erythrocyte progenitors [22, 23], will not be inferred by DeepCT. In addition, in this work, we did not control how the cell types were split between training and validation sets. This may lead to inflated performance when 2 very similar cellular subtypes appear in train and validation datasets. On the other hand, there is currently no “ground true” broad classification of ENCODE cell types that would reflect their epigenetic similarity. The best classification we were able to find (presented in Supplementary Fig. S1) is derived using the ENCODE cell ontology database, reflecting anatomic rather than epigenetic relationships between different cell types. In fact, we hope that the latent space of cell states inferred by DeepCT can be used to build the best epigenetic classification of human cells in the future, although there are some limitations of this approach discussed below.

Second, in this work we employed multiple epigenetic profiles of heterogeneous tissues, composed of different cell types. Applying DeepCT to more pure single-cell datasets can extend cell type classification and may help to solve an important challenge of learning missing modalities from single-cell data.

Third, although we show that cell type representations learned by the DeepCT model are meaningful, we emphasize that these representations include a component associated with technical biases of experiments. Indeed, cell state embedding is learned to explain all dependencies between the measured epigenetic profile and sequence composition specific for a particular cell type. This specificity may originate not only from biological ground but also from poor quality of experimental conditions specific for this cell type. Of course, with a large number of experimental datasets available for 1 cell type (ideally obtained in different laboratories to reduce batch effect), this factor would play a lesser role in resulting embeddings.

Finally, we note that performance of the DeepCT model for some tracks and some sequences was low, although in other cases, we obtained high performance scores. In particular, distribution of broad histone marks was poorly predicted, most probably because longer sequence context is required for accurate inference. We expect that performance of DeepCT can be further improved using more powerful neural network architectures (such as transformer layers [9]), including information about long-range regulation [10] and more epigenetic datasets.

## Methods

### Data collection

#### ENCODE data collection

We downloaded reprocessed ENCODE datasets from EpiMap [11,24]. We used *P* value rather than fold-change as a signal measure, motivated by suggestions from [11]. The complete datasets contained 3,026 tracks (i.e., cell type/epigenetic feature combinations). For model training, we excluded tracks with low-quality tracks (the list was downloaded from supplementary information available in [11]). The resulting dataset includes 2,629 tracks describing 40 epigenetic features (41 if CAGE data are included; see below) characterizing 794 human cell types.

To convert the quantitative *P* value track into qualitative peaks positions, we used an empirical threshold of negative log of *P* value equal to 4.4 (i.e., *P* value below 10^−4.4^). We defined peaks as 150-bp genomic intervals centered around genomic positions where the signal exceeds the threshold. The threshold value and peak length were chosen to match peak positions reported by standard ENCODE processing pipelines. Overlapping peaks were merged, and only those intervals where at least 1 peak was detected were kept in the dataset. For training the quantitative model, we logged data and clipped values using thresholds −1.0…4.0.

#### CAGE dataset collection and processing

We used metadata from the GTRD [25] to match ENCODE with FANTOM5 [26]. Primarily we used *cell_id* identifier from the GTRD, which uniquely describes cell type; in addition, we manually screened results to ensure the same origin and treatment status, removed fetal samples (as expression and epigenetic status may rapidly change during the development and there is no way to ensure a proper match between samples from different organisms even at exact same-day age—which by itself almost never matches between these databases), and corrected annotation mistakes. We downloaded alignments for chosen experiments from [27]; data from biological replicates were merged together. Last, we connected FANTOM5 experiments to EpiMap's *BSSID* identifiers, which describe cell type, age, and treatment status; in case of 1-to-many and many-to-many relationships, we used random assignment to create pairs. The resulting dataset comprised 99 pairings.

To obtain coverage track, we processed alignments with DeepTools bamCoverage using RPKM normalization, a bin size of 10, a smoothing window size of 50, and a minimum mapping quality threshold of 1. For training, we logged data and clipped values using thresholds −7.0…2.0. Note that we used CAGE data only for quantitative models.

### Machine learning models architecture

#### Models inputs and targets

We constructed CNN models with 2 inputs: a sequence input, accepting a 1-hot encoded 1,000-bp DNA sequence (shape 4 × 1,000), and a cell type input, accepting a 1-hot encoded cell type (shape 1 × N_cell_types). We defined a 200-bp region in the center of the input sequence as a target region and constructed the model's target as follows:

(a) For qualitative models, we used binary labels 1 or 0; label 1 indicates that in the specified cell type and epigenetic feature combination, there is the epigenetic feature peak overlapping the selected 200-bp region by at least 50%.
(b) For quantitative models, we used the maximal feature signal value computed across the target 200-bp region for all data except CAGE. For CAGE data, we used the sum of signals.

To sample sequences, we filtered out genomic intervals where no peak of target features is present. We extended the remaining intervals by 30 bp from both sides (to add samples with no peaks) and drew coordinates of centers of samples (sampling points) using uniform distribution. The sampling points were set apart by at least 200 bp, so that their targets would not overlap one another.

For a specific sequence input, we performed a forward pass of the model for all cell types at once to reduce computation load (see below). Thus, the model returned *N_cell_types* × *N_features* values, with each value corresponding to a combination of cell type and epigenetic feature. It is important to note that even if some cell type/epigenetic feature combinations were not experimentally measured, the model output the prediction for these combinations, although these values did not contribute to the model's loss (see below).

#### Models architecture

The models process the DNA sequence by a convolution tail, which outputs sequence embedding of shape 1 × 256. The layers specification is in Supplementary Note 1.

The cell state tail maps a 1-hot encoded cell type from the dimension R^N_cell_types^ to the dimension of cell type embeddings R^emb_length^ using a linear transformation (Supplementary Note 1). We set *emb_length* to 32, which matches the number of cell type groups (based on ENCODE classification) used in this study.

To predict cell type–specific outputs, we concatenated sequence and cell type embeddings and passed the result to the model's tail, which consists of 8 fully connected layers (Supplementary Note 1). We set the number of the last layer's outputs equal to the number of epigenetic features; thus, for each sequence and cell type inputs, we obtained predictions of all epigenetic features.

To reduce computational load, for each sequence, we computed sequence embedding only once and then passed it to the model's head concatenated with different cell type embeddings. Thus, for each sequence, we obtained *N_cell_types* × *N_features* predictions.

After the first trials, we found that predicting data for unseen sequences is substantially more challenging than for unseen cell types (see Results for detail). These results fit with our observation that the portion of the variance in epigenetic data explained by sequence diversity is several times higher than the portion explained by cell type diversity. Motivated by these data, we decided to split the model's head to provide 2 outputs: one providing sequence-specific prediction and another responsible for the cell type–specific part of the prediction.

Let us consider a dataset of *N* cell types characterized by *K* epigenetic tracks. For the genomic position *s*, we aim to predict a set of target values *t_n,k,s_*, where *n* is a cell type index (*n = 1..N*) and *k* is an epigenetic feature index (*k = 1..K*). We defined sequence-specific mean feature positional information (MPI) as *M_k,s_*:


\begin{equation*} {M}_{k,s} = \frac{1}{N}\sum {{t}_{n,k,s},{\mathrm{ }}n{\mathrm{ }} = {\mathrm{ }}1..N} \end{equation*}

and cell type–specific deviations as *D_n,k,s_*:


\begin{equation*} {D}_{n,k,s} = {\mathrm{ }}{t}_{n,k,s} - {\mathrm{ }}{M}_{k,s} \end{equation*}

Thus, *M_k,s_* is the mean feature value, which is invariant across cell types and depends on sequence only; *D_n,k,s_* is cell type–specific deviation, and it depends on cell type and shows how the particular cell type differs from average.

We used 8 fully connected layers with final output shape (1 × N_features) to predict *M* from sequence embedding. Similarly, we used 8 fully connected layers with final output shape *N_cell_types* × *N_features* to predict *D* from concatenated sequence and cell type embeddings. Knowing *M* and *D*, we were able to reconstruct original targets simply by adding up *t_n,k,s_ = D_n,k,s_ + M_k,s_*.

### Computing loss function

We used binary cross-entropy loss for qualitative models and Mean Squared Error(MSE) loss for quantitative models. For a model that outputs *M* and *D* values, we measured MSE loss as


\begin{equation*} loss{\mathrm{ }} = {\mathrm{ }}a*MSE\left( M \right){\mathrm{ }} + {\mathrm{ }}\left( {1 - a} \right)*MSE\left( D \right) \end{equation*}

We found that the *a* value equal to 0.0002 results in optimal performance. Interestingly, the main component of loss, in this case, is cell type specific. Using *a* ∼ 0.5 significantly lowered performance metrics.

The model predicts target values for all cell type/feature combinations; however, for many combinations, there was no experimentally measured data, and therefore loss function cannot be computed for outputs representing these combinations. Thus, only measured targets contribute to the loss. Similarly, we used only a subset of cell type/feature combinations that have experimentally measured data to compute *M* and *D* values.

### Training models

We trained models for 20 epochs using the Tesla V100 16 GB GPU, with an initial learning rate set to 0.0001 and updated during training using a cosine annealing schedule, which we found to give the best model performance. We used a selene-based framework for model training [28].

### Evaluation of models

#### Train and validation split

##### Unseen sequence prediction benchmark

For the unseen sequence prediction benchmark, we split our data by chromosomes, which prevents data leaks between overlapping regions [29]. We used 2 chromosomes for validation, 2 chromosomes for evaluation, and the rest of the genome to train models. Chromosome Y was excluded from all analyses.

##### Unseen cell type prediction benchmark

For an unseen cell type prediction benchmark, we wanted to see how well our model can differentiate between cell types. We did so by evaluating how well it can predict values for unseen combinations of cell types and sequences. We left out 2 chromosomes for model evaluation and split the rest of the chromosomes into several (3, 5, or 10) nonoverlapping folds. We also split a set of cell types into a corresponding number of nonoverlapping sets. We then chose to leave some (3, 5, or 10 respectively) pairs of these sequence and cell type subsets out of training for validation; the rest were kept for training, as demonstrated in Fig. 3A. Note that it would not be enough to simply leave some cell types out of training for validation, as the model would have no data to learn anything about these cell types. However, if we leave out pairs of (cell type, sequence), each cell type is still present in training, and thus we can evaluate whether the model's predictions are cell specific or only sequence specific within sequence folds.

To ensure that all cell type folds contain the same number of tracks, we chose the largest clique from the graph of measured tracks shown in Fig. 1C and trained our models on a subset of tracks given by this clique. This left us with 905 tracks (181 cell types and 5 features).

To select tracks for the unseen track prediction benchmark, we performed the following steps. First, we filtered out all data for cell types represented by fewer than 3 features and for all features with fewer than 3 cell types experimentally profiled. Next, we removed all cell types with treatment. Finally, we aimed to remove 10% of tracks from the training dataset so that these tracks’ data could be predicted using the remaining tracks. For this aim, we defined the track's predictability: measurement of feature *X* in cell type *I* is predictable if and only if there is a cell feature *Y* and cell type *II* satisfying the conditions:

(1) Feature *Y* is measured in cell types *I* and *II*.
(2) Feature *X* is measured in cell type *II*.

We next randomly selected 10% of the tracks so that each of them could be predicted (based on the definition above) using the remaining tracks.

#### Performance metrics

To evaluate the qualitative model, we used the average precision (AP) metric. We pick this metric because it is robust for imbalanced data with numerous negative samples, which is the case for epigenetic datasets (see, e.g., comparison presented in [30]). To evaluate the quantitative model, we used the *r*^2^ metric. To compare results obtained by quantitative and qualitative models, we obtained peak positions from predicted quantitative signal using the same threshold as for experimental data and computed AP scores for the quantitative model. If other was not indicated, all metrics were averaged across features and cell types.

#### Simons Simplex Collection dataset processing and analysis of genomic variant effects

We have retrieved a set of variants discovered via whole-genome sequencing of 1,902 quartet families from the Simons Simplex Collection (Supplementary Table 2 in [12]). Only nonindel proband variants in the 50-kbp proximity of protein-coding genes' transcription starts (as per GENCODEv38) were used. The dataset was lifted over from the GRCh38 to GRCh37 human reference genome, resulting in 50,155 unique variants. We also retrieved another set of variants found independently in 1,790 families from the same collection (Supplementary Table 1 in [13]). These were processed by the same rules except for lifting over (as coordinates there were already on GRCh37), resulting in 28,869 unique variants.

Variants were used to modify the sequence-tail input of DeepCT (RRID:SCR_023302). We used the DeepCT MPI model to infer signals of 40 epigenetic features in 794 cell types for each reference and alternative allele. We then subtracted predictions obtained for the alternative allele from predictions obtained for the reference allele and defined the obtained difference as the cell type–specific *effect* of the variant on the epigenetic feature. We next found a maximum absolute effect across all cell types and features for each genomic variant. Analysis of transcription factors motifs was performed using HOCOMOCO online tools [18].

#### DeepCT validation analysis on GTEx data

We used GTEx v7 data to select variants significantly associated with gene expression [19]. The GTEx portal provides permutation analysis results, where significant variant–gene associations (1 variant per gene) are determined for each tissue (GTEx Consortium 2015). The GTEx consortium collected expression data for 48 tissues. However, for our analysis, we used information from only 22 tissues, which definitely corresponded to the cell state tissues (Supplementary Table S1).

For each of such 22 tissues in the GTEx data, we selected top 50 significant variant–gene associations. In total, we have 584 variants, including specific variants for tissues and common variants for several tissues (up to 9 tissues). So, for each tissue from this list of variants, we can select significant and nonsignificant variants (Supplementary Table S2).

Next, we used the quantitative DeepCT model to predict functional significance of the selected variants. For this, we derived the following importance score: (i) make a prediction for reference and alternative alleles indicated in GTEx summary statistics and (ii) get the subtraction between alternative and reference scores and use it as the DeepCT functional significance score. After that, the DeepCT scores for each SNP were averaged over cell types of the same tissue. Scores for only 11 features with average precision greater than 0.3 and number of samples greater than 10 were included in the analysis: POLR2A, H3K27ac, H2AFZ, CTCF, SMC3, RAD21, ATAC-seq, H3K9ac, H3K4me2, DNase-seq, and H3K4me3 (Fig. 3D, E).

Taking into account that the effect of variants on expression level could be positive or negative, to get comparable distributions, we multiplied the DeepCT score on the sign of variant effect from GTEx summary statistics. Variants with opposite pleiotropic effects on gene expression were excluded. Finally, we applied a *t*-test for DeepCT scores between significant and nonsignificant variants for each tissue and selected features. We adjusted the *t*-test *P* value for multiple comparisons based on the number of tissues (*N* = 22), but not the number of features, since selected features are correlated. Thus, *t*-test results with *P* < 0.0023 were considered significant.

## Availability of Source Code and Requirements

Project name: DeepCT

Project homepage: https://github.com/AIRI-Institute/DeepCT

Operating system(s): Linux

Programming language: Python

Other requirements: numpy (1.20.1), torch (1.8.1), torchvision (0.9.1), pyBigWig (0.3.17), selene (https://github.com/AIRI-Institute/selene)

License: Apache License 2.0.

Any restrictions to use by nonacademics: Nothing except that mentioned in Apache License 2.0.

RRID: SCR_023302

## Additional Files

**Supplementary Table S1**. Number of significant and nonsignificant variants included in analysis for selected tissues.

**Supplementary Table S2**. The *t*-test results between DeepCT scores for significant and nonsignificant variants per tissues and features. Significant results are in bold.

**Supplementary Fig. S1**. Cell state embedding projections from 32-dimensional latent space of the model into a 2-dimensional PCA axis. Background points show distribution of embeddings for all cell types, whereas colored dots correspond to the specific cell types according to the subplot title.

**Supplementary Fig. S2**. DeepCT performance in unseen track benchmark. Same as Fig. 3D, but using *r*^2^ metrics.

**Supplementary Fig. S3**. Projections of embeddings of the same cell types as in Fig. 2C after random shuffling of cell type labels.

**Supplementary Fig. S4**. Saliency maps for 1,000 samples (each row corresponds to 1 sample; each column—1 input letter). Red vertical lines show the start and the end of the target interval. Blue vertical lines correspond to positions 200 and 800 in the sequence.

**Supplementary Fig. S5**. Input gradient for each position in the sequence (mean over 1,000 samples). Since the training dataset mostly contains sequences with a 200-bp intersection, 1 sample contains a target window of another sample. Therefore, there is a consistent increase of importance in the 200-bp intervals. There is also a noticeable drop of gradients centered around the [200, 400, 600, 800] positions in the sequence and at the boInput gradient for each position in the sequence (mean over 1,000 samples). Since the training dataset mostly contains sequences with a 200-bp intersection, 1 sample contains a target window of another sample. Therefore, there is a consistent increase of importance in the 200-bp intervals. There is also a noticeable drop of gradients centered around the [200, 400, 600, 800] positions in the sequence and at both ends of the input sequence, which reflects the training strategy. We would expect this effect to be alleviated by sampling training data with less consistency in sample overlap size.

## Abbreviations

AP: average precision; bp: base pair; CNN: convolutional neural network; kbp: kilbase pair; MPI: mean feature positional information; SNV: single-nucleotide variant.

## Supplementary Material

giad015_GIGA-D-22-00103_Original_Submission

giad015_GIGA-D-22-00103_Revision_1

giad015_Response_to_Reviewer_Comments_Original_Submission

giad015_Reviewer_1_Report_Original_Submission Fangfang Yan -- 6/6/2022 Reviewed

giad015_Reviewer_2_Report_Original_Submission Yuwen Liu -- 6/27/2022 Reviewed

giad015_Reviewer_2_Report_Revision_1 Yuwen Liu -- 12/20/2022 Reviewed

giad015_Reviewer_3_Report_Original_Submission Borbala Mifsud -- 7/4/2022 Reviewed

giad015_Supplemental_File

## Data Availability

All supporting data and materials are available in the *GigaScience* GigaDB database [31].
