# Supplementary material for: Cell type–specific interpretation of noncoding variants using deep learning–based methods
Source: Gigascience. 2023 Mar 27;12:giad015. doi: 10.1093/gigascience/giad015 (PMC10041527; doi:10.1093/gigascience/giad015)
Supplement: giad015_Supplemental_File [file giad015_supplemental_file.docx]

**Supplementary information**

**Supplementary Note 1. Model architectures**

1. **Sequence tail of CNN models, layer parameters provided in bracket**

Conv1d(4, 320, kernel_size=8)

ReLU

Conv1d(320, 320, kernel_size=8)

ReLU

MaxPool1d(kernel_size=4, stride=4)

BatchNorm1d(320)

Conv1d(320, 480, kernel_size=8)

ReLU

Conv1d(480, 480, kernel_size=8)

ReLU

MaxPool1d(kernel_size=4, stride=4)

BatchNorm1d(480)

Dropout(p=0.3)

Conv1d(480, 960, kernel_size=8)

ReLU

Conv1d(960, 960, kernel_size=8)

ReLU

BatchNorm1d(960)

Dropout(p=0.3)

Linear (42240, 256)

1. **Cell type tail of the model**

Linear (N_cell_types, 32)

1. **Model’s head (same architecture used for regressor and classified). We used embedding_length equal 288 (concatenated sequence and cell type embeddings) or 256 (sequence embedding)**

Linear(embedding_length, embedding_length)

ReLU

BatchNorm1d(embedding_length)

Linear(embedding_length, embedding_length)

ReLU

Linear(embedding_length, embedding_length)

ReLU

Linear(embedding_length, embedding_length)

ReLU

BatchNorm1d(embedding_length)

Linear(embedding_length, embedding_length)

ReLU

Linear(embedding_length, embedding_length)

ReLU

Linear(embedding_length, embedding_length)

ReLU

BatchNorm1d(embedding_length)

Linear(embedding_length, n_genomic_features)

**Supplementary Table 1. Number of significant and non-significant variants included in analysis for selected tissues.**

| **GTEx tissue** | **Number of significant variant** | **Number of non-significant variant** | **Number of cell states** |
| --- | --- | --- | --- |
| **Adrenal gland** | **136** | **443** | **14** |
| **Tibial artery** | **134** | **445** | **3** |
| **Cerebellum** | **129** | **450** | **3** |
| **Caudate nucleus** | **113** | **466** | **3** |
| **Hippocampus** | **118** | **461** | **3** |
| **Putamen** | **114** | **465** | **1** |
| **Substantia nigra** | **94** | **485** | **2** |
| **Sigmoid colon** | **121** | **458** | **6** |
| **Transverse colon** | **136** | **443** | **4** |
| **Heart left ventricle** | **125** | **454** | **6** |
| **Liver** | **111** | **468** | **8** |
| **Lung** | **137** | **442** | **43** |
| **Skeletal muscle** | **126** | **453** | **3** |
| **Tibial nerve** | **145** | **434** | **3** |
| **Ovary** | **102** | **477** | **4** |
| **Pancreas** | **140** | **439** | **2** |
| **Spleen** | **127** | **452** | **7** |
| **Stomach** | **125** | **454** | **19** |
| **Testis** | **136** | **443** | **3** |
| **Thyroid gland** | **146** | **433** | **4** |
| **Uterus** | **103** | **476** | **1** |
| **Vagina** | **98** | **481** | **2** |

**Supplementary Table 2. T-test results between DeepCT sores for significant and non-significant variants per tissues and features. Significant results are in bold.**

| **GTEx tissue** | **Features** | **t-test** | **P-value** | **Number of positive variants** | **Number of negative variants** |
| --- | --- | --- | --- | --- | --- |
| **Adrenal gland** | **CTCF** | **1,0255** | **3,06E-01** | **136** | **443** |
|  | **DNase-seq** | **1,0393** | **2,99E-01** | **136** | **443** |
|  | **H2AFZ** | **1,3698** | **1,71E-01** | **136** | **443** |
|  | **H3K4me3** | **1,9391** | **5,30E-02** | **136** | **443** |
|  | **POLR2A** | **1,3939** | **1,64E-01** | **136** | **443** |
|  | **H3K27ac** | **1,3462** | **1,79E-01** | **136** | **443** |
|  | **SMC3** | **0,1107** | **9,12E-01** | **136** | **443** |
|  | **RAD21** | **0,9790** | **3,28E-01** | **136** | **443** |
|  | **H3K4me2** | **2,0706** | **3,88E-02** | **136** | **443** |
|  | **ATAC-seq** | **1,0176** | **3,09E-01** | **136** | **443** |
|  | **H3K9ac** | **1,8712** | **6,18E-02** | **136** | **443** |
| **Tibial artery** | **CTCF** | **3,1120** | **1,95E-03** | **134** | **445** |
|  | **DNase-seq** | **1,7498** | **8,07E-02** | **134** | **445** |
|  | **H2AFZ** | **3,8480** | **1,32E-04** | **134** | **445** |
|  | **H3K4me3** | **4,3569** | **1,56E-05** | **134** | **445** |
|  | **POLR2A** | **2,8570** | **4,43E-03** | **134** | **445** |
|  | **H3K27ac** | **3,8661** | **1,23E-04** | **134** | **445** |
|  | **SMC3** | **1,8758** | **6,12E-02** | **134** | **445** |
|  | **RAD21** | **2,0004** | **4,59E-02** | **134** | **445** |
|  | **H3K4me2** | **4,4873** | **8,71E-06** | **134** | **445** |
|  | **ATAC-seq** | **2,9044** | **3,82E-03** | **134** | **445** |
|  | **H3K9ac** | **4,6649** | **3,84E-06** | **134** | **445** |
| **Cerebellum** | **CTCF** | **0,8967** | **3,70E-01** | **129** | **450** |
|  | **DNase-seq** | **1,1221** | **2,62E-01** | **129** | **450** |
|  | **H2AFZ** | **1,5615** | **1,19E-01** | **129** | **450** |
|  | **H3K4me3** | **2,3615** | **1,85E-02** | **129** | **450** |
|  | **POLR2A** | **2,0870** | **3,73E-02** | **129** | **450** |
|  | **H3K27ac** | **2,1241** | **3,41E-02** | **129** | **450** |
|  | **SMC3** | **0,6626** | **5,08E-01** | **129** | **450** |
|  | **RAD21** | **0,9455** | **3,45E-01** | **129** | **450** |
|  | **H3K4me2** | **1,6050** | **1,09E-01** | **129** | **450** |
|  | **ATAC-seq** | **1,2082** | **2,27E-01** | **129** | **450** |
|  | **H3K9ac** | **2,3118** | **2,11E-02** | **129** | **450** |
| **Caudate nucleus** | **CTCF** | **1,9319** | **5,39E-02** | **113** | **466** |
|  | **DNase-seq** | **1,8970** | **5,83E-02** | **113** | **466** |
|  | **H2AFZ** | **2,1991** | **2,83E-02** | **113** | **466** |
|  | **H3K4me3** | **2,5403** | **1,13E-02** | **113** | **466** |
|  | **POLR2A** | **2,6979** | **7,18E-03** | **113** | **466** |
|  | **H3K27ac** | **2,4745** | **1,36E-02** | **113** | **466** |
|  | **SMC3** | **1,6612** | **9,72E-02** | **113** | **466** |
|  | **RAD21** | **1,0447** | **2,97E-01** | **113** | **466** |
|  | **H3K4me2** | **2,6104** | **9,28E-03** | **113** | **466** |
|  | **ATAC-seq** | **1,0956** | **2,74E-01** | **113** | **466** |
|  | **H3K9ac** | **2,2596** | **2,42E-02** | **113** | **466** |
| **Hippocampus** | **CTCF** | **1,4685** | **1,43E-01** | **118** | **461** |
|  | **DNase-seq** | **1,8499** | **6,48E-02** | **118** | **461** |
|  | **H2AFZ** | **2,0911** | **3,70E-02** | **118** | **461** |
|  | **H3K4me3** | **2,5782** | **1,02E-02** | **118** | **461** |
|  | **POLR2A** | **2,4597** | **1,42E-02** | **118** | **461** |
|  | **H3K27ac** | **2,4885** | **1,31E-02** | **118** | **461** |
|  | **SMC3** | **1,1255** | **2,61E-01** | **118** | **461** |
|  | **RAD21** | **1,3870** | **1,66E-01** | **118** | **461** |
|  | **H3K4me2** | **2,3215** | **2,06E-02** | **118** | **461** |
|  | **ATAC-seq** | **1,4486** | **1,48E-01** | **118** | **461** |
|  | **H3K9ac** | **2,4846** | **1,33E-02** | **118** | **461** |
| **Putamen** | **CTCF** | **1,3031** | **1,93E-01** | **114** | **465** |
|  | **DNase-seq** | **3,0282** | **2,57E-03** | **114** | **465** |
|  | **H2AFZ** | **0,5344** | **5,93E-01** | **114** | **465** |
|  | **H3K4me3** | **2,8211** | **4,95E-03** | **114** | **465** |
|  | **POLR2A** | **1,5986** | **1,10E-01** | **114** | **465** |
|  | **H3K27ac** | **2,5222** | **1,19E-02** | **114** | **465** |
|  | **SMC3** | **0,1138** | **9,09E-01** | **114** | **465** |
|  | **RAD21** | **0,5516** | **5,81E-01** | **114** | **465** |
|  | **H3K4me2** | **3,0625** | **2,30E-03** | **114** | **465** |
|  | **ATAC-seq** | **1,6618** | **9,71E-02** | **114** | **465** |
|  | **H3K9ac** | **2,8157** | **5,03E-03** | **114** | **465** |
| **Substantia nigra** | **CTCF** | **0,0408** | **9,67E-01** | **94** | **485** |
|  | **DNase-seq** | **1,5807** | **1,14E-01** | **94** | **485** |
|  | **H2AFZ** | **0,6276** | **5,31E-01** | **94** | **485** |
|  | **H3K4me3** | **2,5840** | **1,00E-02** | **94** | **485** |
|  | **POLR2A** | **1,2908** | **1,97E-01** | **94** | **485** |
|  | **H3K27ac** | **3,2592** | **1,18E-03** | **94** | **485** |
|  | **SMC3** | **-0,6055** | **5,45E-01** | **94** | **485** |
|  | **RAD21** | **-0,0106** | **9,92E-01** | **94** | **485** |
|  | **H3K4me2** | **2,8033** | **5,23E-03** | **94** | **485** |
|  | **ATAC-seq** | **-0,2568** | **7,97E-01** | **94** | **485** |
|  | **H3K9ac** | **2,6949** | **7,25E-03** | **94** | **485** |
| **Sigmoid colon** | **CTCF** | **1,3667** | **1,72E-01** | **121** | **458** |
|  | **DNase-seq** | **1,0747** | **2,83E-01** | **121** | **458** |
|  | **H2AFZ** | **1,5849** | **1,14E-01** | **121** | **458** |
|  | **H3K4me3** | **2,8764** | **4,17E-03** | **121** | **458** |
|  | **POLR2A** | **1,7718** | **7,70E-02** | **121** | **458** |
|  | **H3K27ac** | **1,6064** | **1,09E-01** | **121** | **458** |
|  | **SMC3** | **0,4267** | **6,70E-01** | **121** | **458** |
|  | **RAD21** | **1,4296** | **1,53E-01** | **121** | **458** |
|  | **H3K4me2** | **2,6225** | **8,96E-03** | **121** | **458** |
|  | **ATAC-seq** | **1,8755** | **6,12E-02** | **121** | **458** |
|  | **H3K9ac** | **2,1216** | **3,43E-02** | **121** | **458** |
| **Transverse colon** | **CTCF** | **1,6581** | **9,78E-02** | **136** | **443** |
|  | **DNase-seq** | **0,7593** | **4,48E-01** | **136** | **443** |
|  | **H2AFZ** | **2,0513** | **4,07E-02** | **136** | **443** |
|  | **H3K4me3** | **2,5191** | **1,20E-02** | **136** | **443** |
|  | **POLR2A** | **1,0796** | **2,81E-01** | **136** | **443** |
|  | **H3K27ac** | **1,5811** | **1,14E-01** | **136** | **443** |
|  | **SMC3** | **0,5647** | **5,72E-01** | **136** | **443** |
|  | **RAD21** | **0,8642** | **3,88E-01** | **136** | **443** |
|  | **H3K4me2** | **2,4613** | **1,41E-02** | **136** | **443** |
|  | **ATAC-seq** | **0,7817** | **4,35E-01** | **136** | **443** |
|  | **H3K9ac** | **2,2837** | **2,28E-02** | **136** | **443** |
| **Heart left ventricle** | **CTCF** | **2,8430** | **4,63E-03** | **125** | **454** |
|  | **DNase-seq** | **3,0651** | **2,28E-03** | **125** | **454** |
|  | **H2AFZ** | **2,2855** | **2,26E-02** | **125** | **454** |
|  | **H3K4me3** | **2,8672** | **4,29E-03** | **125** | **454** |
|  | **POLR2A** | **3,2842** | **1,08E-03** | **125** | **454** |
|  | **H3K27ac** | **2,5343** | **1,15E-02** | **125** | **454** |
|  | **SMC3** | **1,9138** | **5,61E-02** | **125** | **454** |
|  | **RAD21** | **1,7982** | **7,27E-02** | **125** | **454** |
|  | **H3K4me2** | **3,6558** | **2,80E-04** | **125** | **454** |
|  | **ATAC-seq** | **1,7325** | **8,37E-02** | **125** | **454** |
|  | **H3K9ac** | **2,6732** | **7,73E-03** | **125** | **454** |
| **Liver** | **CTCF** | **1,1551** | **2,49E-01** | **111** | **468** |
|  | **DNase-seq** | **0,9148** | **3,61E-01** | **111** | **468** |
|  | **H2AFZ** | **1,1837** | **2,37E-01** | **111** | **468** |
|  | **H3K4me3** | **1,8181** | **6,96E-02** | **111** | **468** |
|  | **POLR2A** | **1,5762** | **1,16E-01** | **111** | **468** |
|  | **H3K27ac** | **1,7676** | **7,77E-02** | **111** | **468** |
|  | **SMC3** | **0,5666** | **5,71E-01** | **111** | **468** |
|  | **RAD21** | **0,8858** | **3,76E-01** | **111** | **468** |
|  | **H3K4me2** | **1,5310** | **1,26E-01** | **111** | **468** |
|  | **ATAC-seq** | **0,6152** | **5,39E-01** | **111** | **468** |
|  | **H3K9ac** | **1,7214** | **8,57E-02** | **111** | **468** |
| **Lung** | **CTCF** | **3,7257** | **2,14E-04** | **137** | **442** |
|  | **DNase-seq** | **2,1263** | **3,39E-02** | **137** | **442** |
|  | **H2AFZ** | **2,8693** | **4,26E-03** | **137** | **442** |
|  | **H3K4me3** | **3,3051** | **1,01E-03** | **137** | **442** |
|  | **POLR2A** | **2,9883** | **2,92E-03** | **137** | **442** |
|  | **H3K27ac** | **2,9097** | **3,76E-03** | **137** | **442** |
|  | **SMC3** | **2,4829** | **1,33E-02** | **137** | **442** |
|  | **RAD21** | **2,3939** | **1,70E-02** | **137** | **442** |
|  | **H3K4me2** | **3,6890** | **2,46E-04** | **137** | **442** |
|  | **ATAC-seq** | **2,3087** | **2,13E-02** | **137** | **442** |
|  | **H3K9ac** | **3,6444** | **2,92E-04** | **137** | **442** |
| **Skeletal muscle** | **CTCF** | **1,7149** | **8,69E-02** | **126** | **453** |
|  | **DNase-seq** | **2,4832** | **1,33E-02** | **126** | **453** |
|  | **H2AFZ** | **2,8790** | **4,14E-03** | **126** | **453** |
|  | **H3K4me3** | **3,2022** | **1,44E-03** | **126** | **453** |
|  | **POLR2A** | **2,5318** | **1,16E-02** | **126** | **453** |
|  | **H3K27ac** | **3,0108** | **2,72E-03** | **126** | **453** |
|  | **SMC3** | **1,9501** | **5,17E-02** | **126** | **453** |
|  | **RAD21** | **1,7146** | **8,70E-02** | **126** | **453** |
|  | **H3K4me2** | **3,2495** | **1,22E-03** | **126** | **453** |
|  | **ATAC-seq** | **2,3924** | **1,71E-02** | **126** | **453** |
|  | **H3K9ac** | **3,3967** | **7,29E-04** | **126** | **453** |
| **Tibial nerve** | **CTCF** | **3,5575** | **4,05E-04** | **145** | **434** |
|  | **DNase-seq** | **0,7066** | **4,80E-01** | **145** | **434** |
|  | **H2AFZ** | **3,2022** | **1,44E-03** | **145** | **434** |
|  | **H3K4me3** | **4,3242** | **1,80E-05** | **145** | **434** |
|  | **POLR2A** | **2,2434** | **2,52E-02** | **145** | **434** |
|  | **H3K27ac** | **2,4322** | **1,53E-02** | **145** | **434** |
|  | **SMC3** | **2,1578** | **3,14E-02** | **145** | **434** |
|  | **RAD21** | **3,0429** | **2,45E-03** | **145** | **434** |
|  | **H3K4me2** | **3,5098** | **4,83E-04** | **145** | **434** |
|  | **ATAC-seq** | **2,5481** | **1,11E-02** | **145** | **434** |
|  | **H3K9ac** | **3,9244** | **9,74E-05** | **145** | **434** |
| **Ovary** | **CTCF** | **1,0864** | **2,78E-01** | **102** | **477** |
|  | **DNase-seq** | **0,2740** | **7,84E-01** | **102** | **477** |
|  | **H2AFZ** | **0,7751** | **4,39E-01** | **102** | **477** |
|  | **H3K4me3** | **1,5401** | **1,24E-01** | **102** | **477** |
|  | **POLR2A** | **0,6801** | **4,97E-01** | **102** | **477** |
|  | **H3K27ac** | **0,6807** | **4,96E-01** | **102** | **477** |
|  | **SMC3** | **0,5117** | **6,09E-01** | **102** | **477** |
|  | **RAD21** | **0,8677** | **3,86E-01** | **102** | **477** |
|  | **H3K4me2** | **1,2339** | **2,18E-01** | **102** | **477** |
|  | **ATAC-seq** | **-0,0482** | **9,62E-01** | **102** | **477** |
|  | **H3K9ac** | **1,3835** | **1,67E-01** | **102** | **477** |
| **Pancreas** | **CTCF** | **1,1401** | **2,55E-01** | **140** | **439** |
|  | **DNase-seq** | **3,3625** | **8,23E-04** | **140** | **439** |
|  | **H2AFZ** | **-0,3819** | **7,03E-01** | **140** | **439** |
|  | **H3K4me3** | **3,4402** | **6,23E-04** | **140** | **439** |
|  | **POLR2A** | **3,6620** | **2,73E-04** | **140** | **439** |
|  | **H3K27ac** | **2,1820** | **2,95E-02** | **140** | **439** |
|  | **SMC3** | **2,8644** | **4,33E-03** | **140** | **439** |
|  | **RAD21** | **3,1933** | **1,48E-03** | **140** | **439** |
|  | **H3K4me2** | **3,7599** | **1,87E-04** | **140** | **439** |
|  | **ATAC-seq** | **1,0866** | **2,78E-01** | **140** | **439** |
|  | **H3K9ac** | **2,1805** | **2,96E-02** | **140** | **439** |
| **Spleen** | **CTCF** | **1,7997** | **7,24E-02** | **127** | **452** |
|  | **DNase-seq** | **1,7848** | **7,48E-02** | **127** | **452** |
|  | **H2AFZ** | **1,0043** | **3,16E-01** | **127** | **452** |
|  | **H3K4me3** | **1,9221** | **5,51E-02** | **127** | **452** |
|  | **POLR2A** | **2,4800** | **1,34E-02** | **127** | **452** |
|  | **H3K27ac** | **1,7189** | **8,62E-02** | **127** | **452** |
|  | **SMC3** | **0,8933** | **3,72E-01** | **127** | **452** |
|  | **RAD21** | **1,4185** | **1,57E-01** | **127** | **452** |
|  | **H3K4me2** | **2,0557** | **4,03E-02** | **127** | **452** |
|  | **ATAC-seq** | **1,2590** | **2,09E-01** | **127** | **452** |
|  | **H3K9ac** | **1,8643** | **6,28E-02** | **127** | **452** |
| **Stomach** | **CTCF** | **1,7179** | **8,64E-02** | **125** | **454** |
|  | **DNase-seq** | **1,5519** | **1,21E-01** | **125** | **454** |
|  | **H2AFZ** | **1,6344** | **1,03E-01** | **125** | **454** |
|  | **H3K4me3** | **2,1561** | **3,15E-02** | **125** | **454** |
|  | **POLR2A** | **2,8378** | **4,70E-03** | **125** | **454** |
|  | **H3K27ac** | **1,5369** | **1,25E-01** | **125** | **454** |
|  | **SMC3** | **0,8847** | **3,77E-01** | **125** | **454** |
|  | **RAD21** | **2,0035** | **4,56E-02** | **125** | **454** |
|  | **H3K4me2** | **2,3447** | **1,94E-02** | **125** | **454** |
|  | **ATAC-seq** | **1,7228** | **8,55E-02** | **125** | **454** |
|  | **H3K9ac** | **2,2756** | **2,32E-02** | **125** | **454** |
| **Testis** | **CTCF** | **1,1150** | **2,65E-01** | **136** | **443** |
|  | **DNase-seq** | **0,0907** | **9,28E-01** | **136** | **443** |
|  | **H2AFZ** | **1,5869** | **1,13E-01** | **136** | **443** |
|  | **H3K4me3** | **2,1916** | **2,88E-02** | **136** | **443** |
|  | **POLR2A** | **1,7424** | **8,20E-02** | **136** | **443** |
|  | **H3K27ac** | **2,0167** | **4,42E-02** | **136** | **443** |
|  | **SMC3** | **0,0098** | **9,92E-01** | **136** | **443** |
|  | **RAD21** | **0,3693** | **7,12E-01** | **136** | **443** |
|  | **H3K4me2** | **2,3956** | **1,69E-02** | **136** | **443** |
|  | **ATAC-seq** | **0,7187** | **4,73E-01** | **136** | **443** |
|  | **H3K9ac** | **2,4409** | **1,50E-02** | **136** | **443** |
| **Thyroid gland** | **CTCF** | **1,3385** | **1,81E-01** | **146** | **433** |
|  | **DNase-seq** | **0,8477** | **3,97E-01** | **146** | **433** |
|  | **H2AFZ** | **1,7434** | **8,18E-02** | **146** | **433** |
|  | **H3K4me3** | **1,6690** | **9,57E-02** | **146** | **433** |
|  | **POLR2A** | **1,0352** | **3,01E-01** | **146** | **433** |
|  | **H3K27ac** | **1,3776** | **1,69E-01** | **146** | **433** |
|  | **SMC3** | **0,2887** | **7,73E-01** | **146** | **433** |
|  | **RAD21** | **0,4647** | **6,42E-01** | **146** | **433** |
|  | **H3K4me2** | **2,0616** | **3,97E-02** | **146** | **433** |
|  | **ATAC-seq** | **1,0405** | **2,99E-01** | **146** | **433** |
|  | **H3K9ac** | **1,7905** | **7,39E-02** | **146** | **433** |
| **Uterus** | **CTCF** | **0,3198** | **7,49E-01** | **103** | **476** |
|  | **DNase-seq** | **-0,0310** | **9,75E-01** | **103** | **476** |
|  | **H2AFZ** | **0,7506** | **4,53E-01** | **103** | **476** |
|  | **H3K4me3** | **1,7064** | **8,85E-02** | **103** | **476** |
|  | **POLR2A** | **0,2734** | **7,85E-01** | **103** | **476** |
|  | **H3K27ac** | **2,0113** | **4,48E-02** | **103** | **476** |
|  | **SMC3** | **0,4024** | **6,88E-01** | **103** | **476** |
|  | **RAD21** | **0,2606** | **7,95E-01** | **103** | **476** |
|  | **H3K4me2** | **1,3353** | **1,82E-01** | **103** | **476** |
|  | **ATAC-seq** | **-0,4719** | **6,37E-01** | **103** | **476** |
|  | **H3K9ac** | **1,4996** | **1,34E-01** | **103** | **476** |
| **Vagina** | **CTCF** | **1,7937** | **7,34E-02** | **98** | **481** |
|  | **DNase-seq** | **-0,7283** | **4,67E-01** | **98** | **481** |
|  | **H2AFZ** | **2,1172** | **3,47E-02** | **98** | **481** |
|  | **H3K4me3** | **2,3240** | **2,05E-02** | **98** | **481** |
|  | **POLR2A** | **0,9272** | **3,54E-01** | **98** | **481** |
|  | **H3K27ac** | **1,1342** | **2,57E-01** | **98** | **481** |
|  | **SMC3** | **-1,0172** | **3,10E-01** | **98** | **481** |
|  | **RAD21** | **-0,7006** | **4,84E-01** | **98** | **481** |
|  | **H3K4me2** | **1,5733** | **1,16E-01** | **98** | **481** |
|  | **ATAC-seq** | **0,9268** | **3,54E-01** | **98** | **481** |
|  | **H3K9ac** | **1,7941** | **7,33E-02** | **98** | **481** |

**Supplementary Figure 1.** *Cell state embeddings projections from 32-dimensional latent space of the model into 2-dimension PCA axis. Background points show distribution of embeddings for all cell types, whereas colored dots correspond to the specific cell types according to the subplot title.*


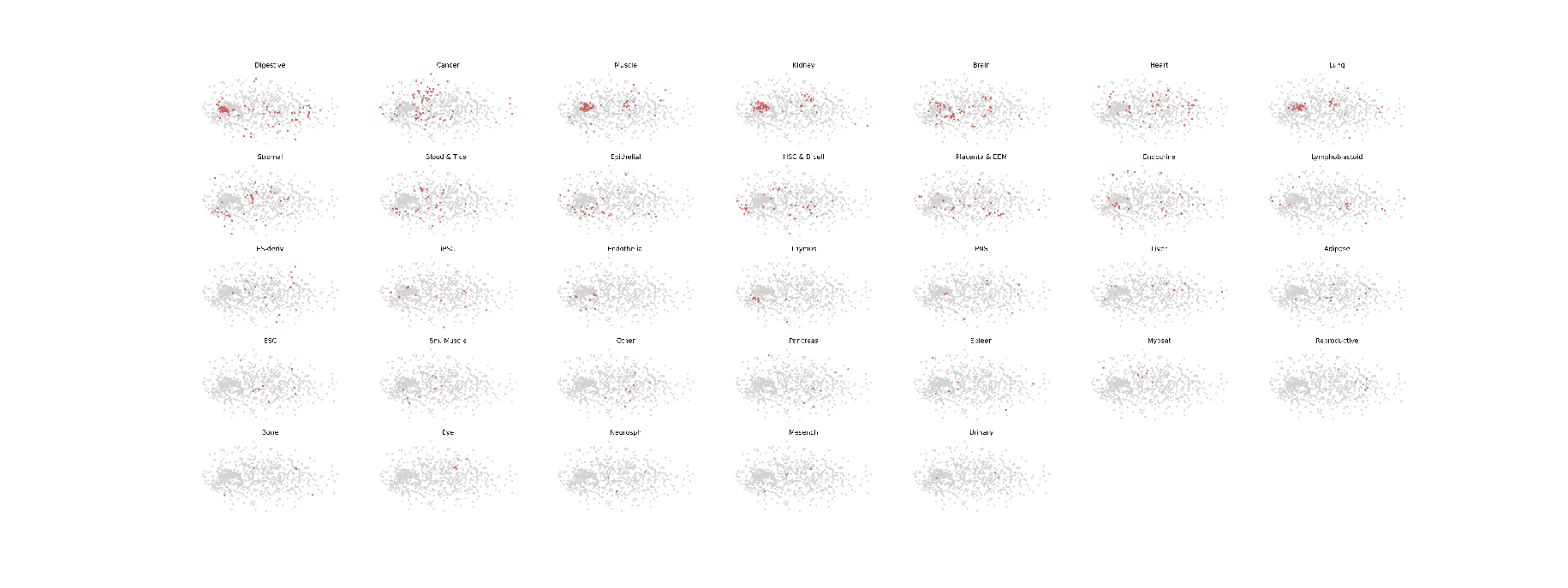


**Supplementary Figure 2.** *DeepCT performance in unseen track benchmark. Same as Fig. 3, D, but using r2 metrics.*

**
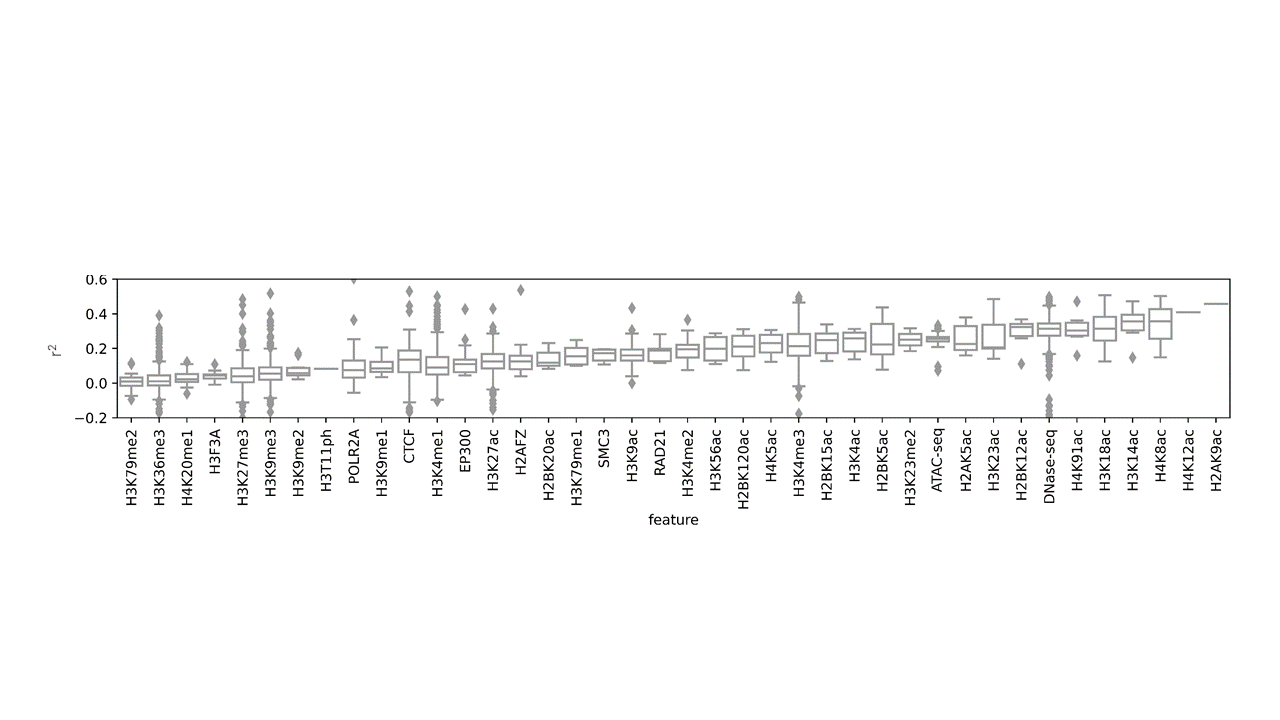
**

**Supplementary Figure 3. Projections of embeddings of the same cell types as in Fig. 2C after random shuffling of cell type labels.**

**
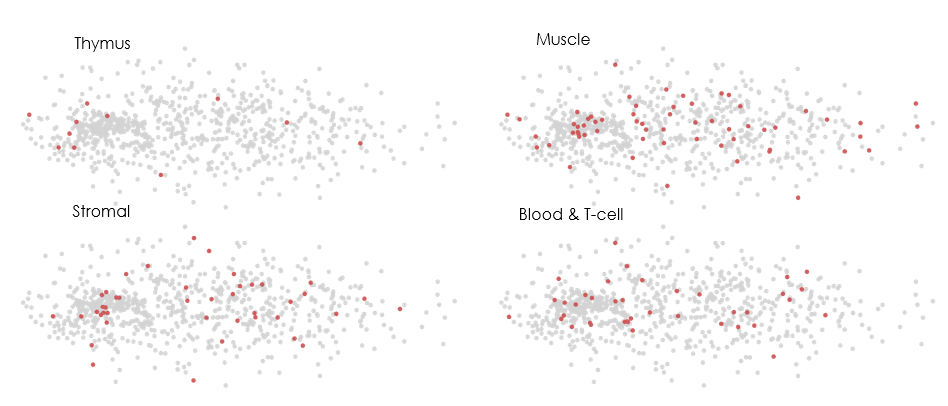
**

**Supplementary Figure 4. Saliency maps for 1000 samples (each row corresponds to one sample, each column — one input letter). Red vertical lines show the start and the end of the target interval. Blue vertical lines correspond to positions 200 and 800 in the sequence.**

**
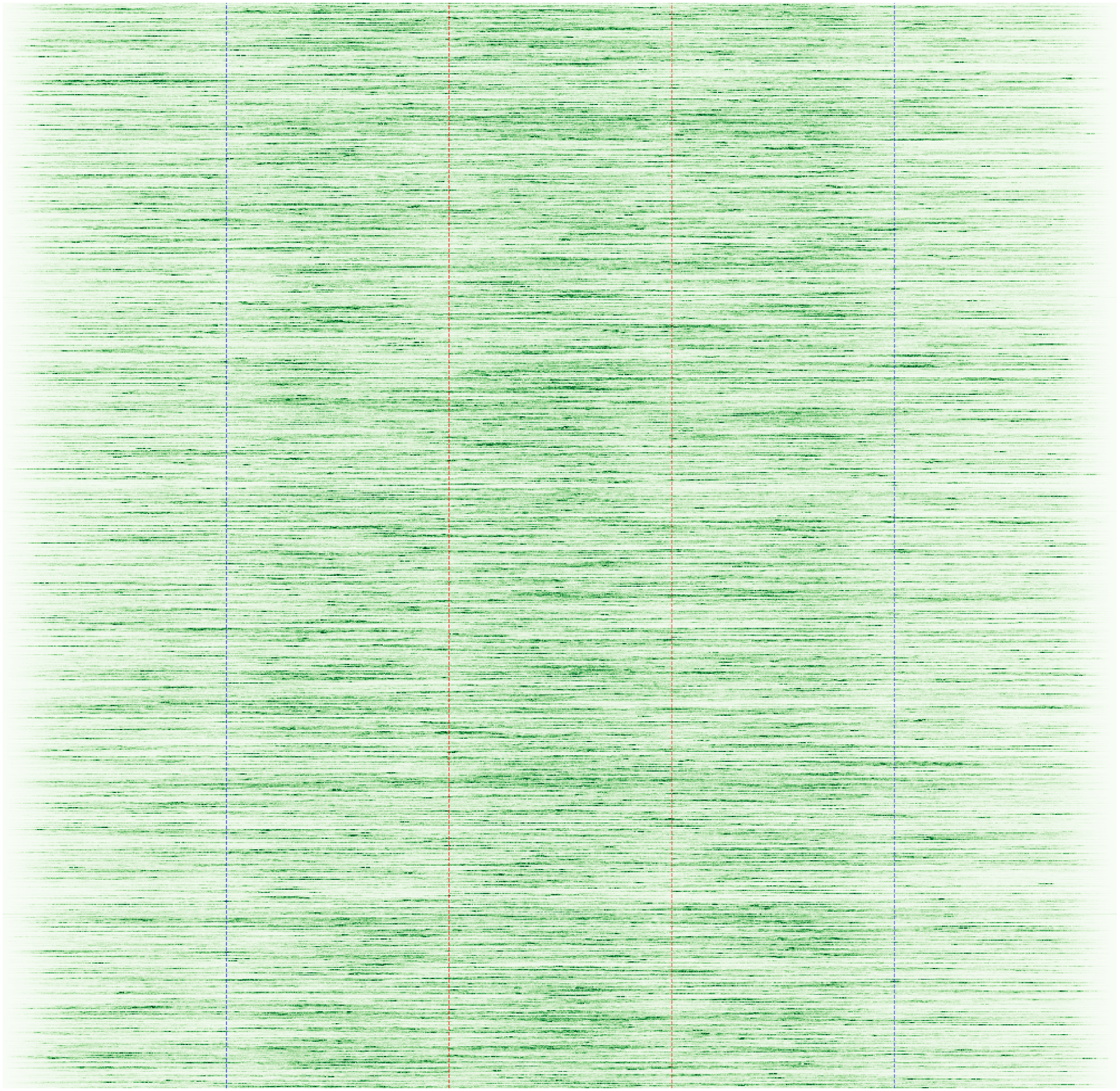
**

**Supplementary Figure 5.** *Input gradient for each position in the sequence (mean over 1000 samples). Since the training dataset mostly contains sequences with 200bp intersection, one sample contains a target window of another sample. Therefore, there is a consistent increase of importance in the 200bp intervals. There is also a noticeable drop of gradients centered around the [200, 400, 600, 800] positions in the sequence and at boInput gradient for each position in the sequence (mean over 1000 samples). Since the training dataset mostly contains sequences with 200bp intersection, one sample contains a target window of another sample. Therefore, there is a consistent increase of importance in the 200bp intervals. There is also a noticeable drop of gradients centered around the [200, 400, 600, 800] positions in the sequence and at both ends of the input sequence, which reflects the training strategy. We would expect this effect to be alleviated by sampling training data with less consistency in sample overlap size.*

*th ends of the input sequence, which reflects the training strategy. We would expect this effect to be alleviated by sampling training data with less consistency in sample overlap size.*

**
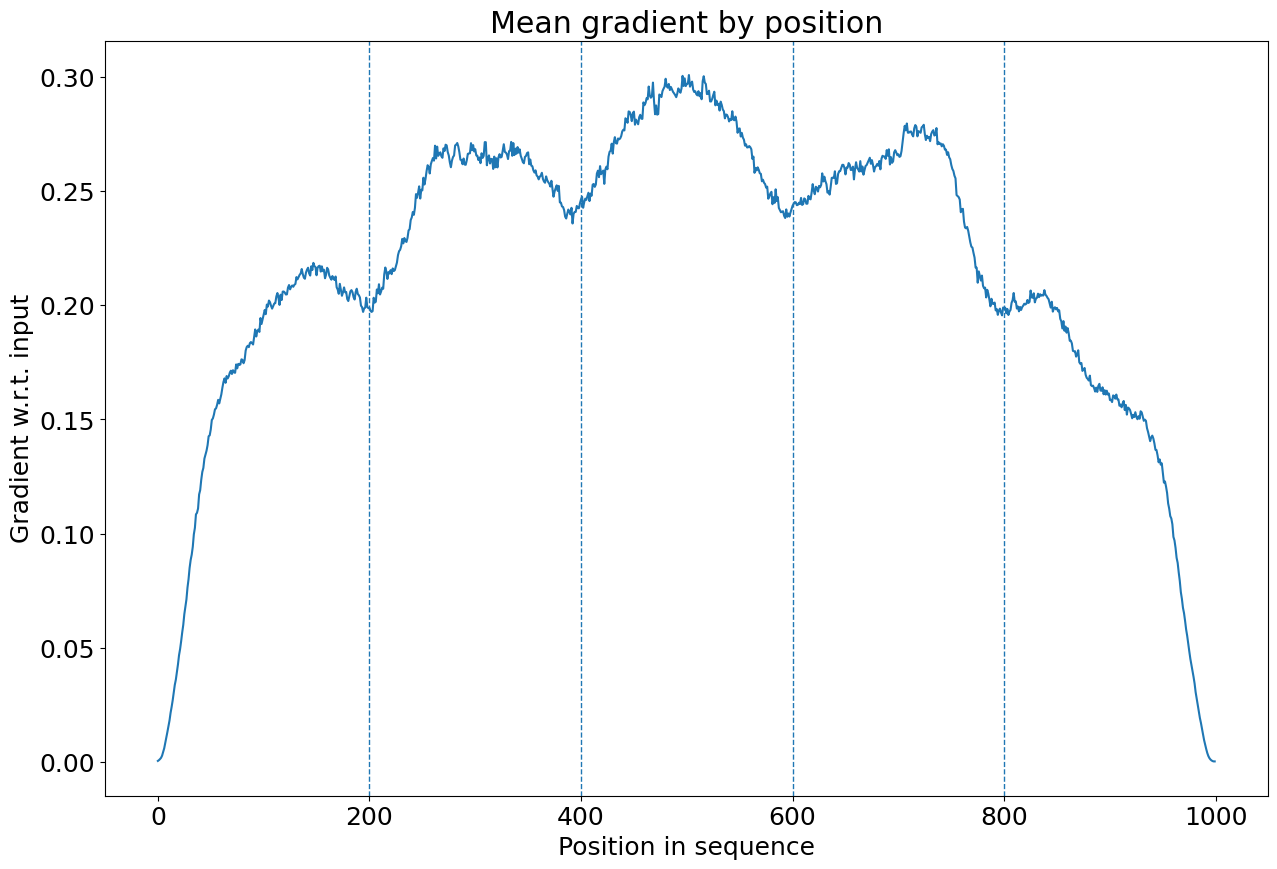
**

**Supplementary Video 1.** *Supplementary video showing how model fits embeddings for three kidney and three muscle cell types. Each frame shows projection of embeddings from 32-dimensional latent space of the model into 2-dimension PCA axis.*
