# Supplementary material for: Cell type–specific interpretation of noncoding variants using deep learning–based methods
Source: Gigascience. 2023 Mar 27;12:giad015. doi: 10.1093/gigascience/giad015 (PMC10041527; doi:10.1093/gigascience/giad015)
Supplement: giad015_Response_to_Reviewer_Comments_Original_Submission [file giad015_response_to_reviewer_comments_original_submission.pdf]

Author's Response To Reviewer Comments

Close

We have included multiple figures to supplement our responses to reviewer and editor comments. Please follow Supplementary File "Rebuttal Letter" to view it.

Close
