# Supplementary material for: Cell type–specific interpretation of noncoding variants using deep learning–based methods
Source: Gigascience. 2023 Mar 27;12:giad015. doi: 10.1093/gigascience/giad015 (PMC10041527; doi:10.1093/gigascience/giad015)
Supplement: giad015_Reviewer_1_Report_Original_Submission — Fangfang Yan -- 6/6/2022 Reviewed [file giad015_reviewer_1_report_original_submission.pdf]

Reviewer Report

Title: Cell type-specific interpretation of noncoding variants using deep learning-based methods

Version: Original Submission **Date:** 6/6/2022

Reviewer name: Fangfang Yan

Reviewer Comments to Author:

In this manuscript, Sindeeva and colleagues describe a novel neural network-based algorithm, DeepCT, to cluster epigenetically similar cell types and infer unmeasured epigenetic features, which then can be used to interpret non-coding variants.
The manuscript is well structured and well written, it is potentially interesting to a broad readership. Yet, the algorithm itself in the manuscript lacks rigor and thoroughness.
Major points:
1. Lack of comparison with competing methods
2. As the authors state themselves in the results and discussion, the performance of DeepCT among some features is very low, such as H3K9 and H4K20 monomethylation. Could authors add more discussion and explanations of this almost zero average precision?
3. The authors said "statistically higher" or "outperforms" in a lot of statements but no statical test results. For example, on page 8, the authors write: "This analysis confirmed that average cosine similarity for embeddings representing cell types from the same tissue was significantly higher than for embeddings of randomly selected cell types". On page 9, "we note that this baseline has performance metrics substantially higher than expected in random (baseline AP=0.417)."
4. On page 8, the authors write "we show co-localization of muscle cells, as well as co-localization of digestive cells (Fig. 2C)". However, Figure 2C looks not quite convincing.
Minor points:
1. Providing high-resolution vector-friendly figures will help a lot. I can barely see the content of the figure in the current version.
2. A jupyter notebook tutorial on the Github repo would be helpful for users to apply DeepCT quickly.

Methods

Are the methods appropriate to the aims of the study, are they well described, and are necessary controls included? Choose an item.

Conclusions

Are the conclusions adequately supported by the data shown? Choose an item.

Reporting Standards

Does the manuscript adhere to the journal's guidelines on [minimum standards of reporting?] Choose an item.

Choose an item.

Statistics

Are you able to assess all statistics in the manuscript, including the appropriateness of statistical tests used? Choose an item.

Quality of Written English

Please indicate the quality of language in the manuscript: Choose an item.

Declaration of Competing Interests

Please complete a declaration of competing interests, considering the following questions:

- Have you in the past five years received reimbursements, fees, funding, or salary from an organisation that may in any way gain or lose financially from the publication of this manuscript, either now or in the future?
- Do you hold any stocks or shares in an organisation that may in any way gain or lose financially from the publication of this manuscript, either now or in the future?
- Do you hold or are you currently applying for any patents relating to the content of the manuscript?
- Have you received reimbursements, fees, funding, or salary from an organization that holds or has applied for patents relating to the content of the manuscript?
- Do you have any other financial competing interests?
- Do you have any non-financial competing interests in relation to this paper?

If you can answer no to all of the above, write 'I declare that I have no competing interests' below. If your reply is yes to any, please give details below.

I declare that I have no competing interests.

I agree to the open peer review policy of the journal. I understand that my name will be included on my report to the authors and, if the manuscript is accepted for publication, my named report including any attachments I upload will be posted on the website along with the authors' responses. I agree for my report to be made available under an Open Access Creative Commons CC-BY license (http://creativecommons.org/licenses/by/4.0/). I understand that any comments which I do not wish to be included in my named report can be included as confidential comments to the editors, which will not be published.

Choose an item.

To further support our reviewers, we have joined with Publons, where you can gain additional credit to further highlight your hard work (see: https://publons.com/journal/530/gigascience). On publication of this paper, your review will be automatically added to Publons, you can then choose whether or not to claim your Publons credit. I understand this statement.

Yes Choose an item.
