# Supplementary material for: Cell type–specific interpretation of noncoding variants using deep learning–based methods
Source: Gigascience. 2023 Mar 27;12:giad015. doi: 10.1093/gigascience/giad015 (PMC10041527; doi:10.1093/gigascience/giad015)
Supplement: giad015_Reviewer_2_Report_Original_Submission — Yuwen Liu -- 6/27/2022 Reviewed [file giad015_reviewer_2_report_original_submission.pdf]

Reviewer Report

Title: Cell type-specific interpretation of noncoding variants using deep learning-based methods

Version: Original Submission **Date:** 6/27/2022

Reviewer name: Yuwen Liu

Reviewer Comments to Author:

The manuscript entitled "Cell type-specific interpretation of noncoding variants using deep learning-based methods" interpreted the non-coding genomic variants by integrating the single-cell epigenetic profiles with the convolution neural network. The author found the CNN can capture the cell type-specific properties and generate a biologically meaningful cell state representation by embedding the cell to the latent space. In general, the architecture of the convolution neural network is novel, and, to a certain extent, the model may be helpful for improving our understanding of genomic non-coding variant effects at single-cell level.

Major comments:

1. In Figure1C the author intended to quantify how often unmeasured epigenetic marks can be inferred from available profiles. Although, in fact, the modification of the epigenetic marks is correlated and sometimes colocated in the genome (Ernst and Kellis 2015). However, the connected graph is not a piece of strong and solid evidence or data for quantify the predictive ability of the epigenetic marks. They should provide other compelling evidences or undertake more analysis.
2. The author used an empirical p-value threshold to detect the peak position along the genome. The definition of the peaks for epigenetic mark is crucial for the whole study. At least they should plot the distribution of p-value and explain why they choose the empirical threshold of p-value as 4.4 in detail. Furthermore, the false positive outcome of the test should be corrected.
3. Some epigenetic marks present broad modified regions of the genome, the 150 bp DNA sequence may not contain all the sequence determinants for that broad peak. That is may the prediction performance is poor for most of epigenetic marks.
4. In Figure3D and Supplementary Figure 2, the majority of epigenetic marks presented very poor prediction performance. The author should discuss the potential biological reasons that lead to this result and perform some analyses to preclude these confounding factors.
5.The author should scrutinize their data because they also use some epigenetic profiles form heterogeneous tissues which are composed of different cell types. And these heterogeneous profiles may weaken the predictive power of the convolution neural network model and impair the interpretability of the model.
6. The authors only used SSC data to showcase their predictive power in pinpointing potential causal non-coding variants of ASD. I suggest use GWAS data from a wide varieties of complex traits and diseases to generate a more thorough evaluation of the specificity of their prediction. Furthermore, the authors used prediction leveraging signals from 794 cell types in predicting non-coding causal variants for ASD. Including a large number of ASD-irrelevant cell types would likely bring strong noise and make the results hard to interpret. I suggest the authors mask the epigenetic marks of ASD-relevant cell types

(treating these cells as if they do not have available epigenetic data), and then use epigenetic marks from other cell types to predict non-coding variants with high impact on epigenetic marks in ASD-relevant cells. Then use this new prediction to rerun Fig 4A and 4B. Achieving good performance with this new analysis would better demonstrate the core advantage of their new model, i.e., predicting cell-type specific non-coding effects of cell using epigenetic information from other cell types.
Minor comments:
1. The author defined peaks as 150 bp genomic intervals, however, they use 200 bp DNA sequence as the center when preparing the data for the CNN input.
2. The resolution of the figure should be greatly improved.

Methods

Are the methods appropriate to the aims of the study, are they well described, and are necessary controls included? Choose an item.

Conclusions

Are the conclusions adequately supported by the data shown? Choose an item.

Reporting Standards

Does the manuscript adhere to the journal's guidelines on minimum standards of reporting? Choose an item.

Choose an item.

Statistics

Are you able to assess all statistics in the manuscript, including the appropriateness of statistical tests used? Choose an item.

Quality of Written English

Please indicate the quality of language in the manuscript: Choose an item.

Declaration of Competing Interests

Please complete a declaration of competing interests, considering the following questions:

- Have you in the past five years received reimbursements, fees, funding, or salary from an organisation that may in any way gain or lose financially from the publication of this manuscript, either now or in the future?
- Do you hold any stocks or shares in an organisation that may in any way gain or lose financially from the publication of this manuscript, either now or in the future?

- Do you hold or are you currently applying for any patents relating to the content of the manuscript?
- Have you received reimbursements, fees, funding, or salary from an organization that holds or has applied for patents relating to the content of the manuscript?
- Do you have any other financial competing interests?
- Do you have any non-financial competing interests in relation to this paper?

If you can answer no to all of the above, write 'I declare that I have no competing interests' below. If your reply is yes to any, please give details below.

I declare that I have no competing interests

I agree to the open peer review policy of the journal. I understand that my name will be included on my report to the authors and, if the manuscript is accepted for publication, my named report including any attachments I upload will be posted on the website along with the authors' responses. I agree for my report to be made available under an Open Access Creative Commons CC-BY license (http://creativecommons.org/licenses/by/4.0/). I understand that any comments which I do not wish to be included in my named report can be included as confidential comments to the editors, which will not be published.

Choose an item.

To further support our reviewers, we have joined with Publons, where you can gain additional credit to further highlight your hard work (see: https://publons.com/journal/530/gigascience). On publication of this paper, your review will be automatically added to Publons, you can then choose whether or not to claim your Publons credit. I understand this statement.

Yes Choose an item.
