# Supplementary material for: Cell type–specific interpretation of noncoding variants using deep learning–based methods
Source: Gigascience. 2023 Mar 27;12:giad015. doi: 10.1093/gigascience/giad015 (PMC10041527; doi:10.1093/gigascience/giad015)
Supplement: giad015_Reviewer_3_Report_Original_Submission — Borbala Mifsud -- 7/4/2022 Reviewed [file giad015_reviewer_3_report_original_submission.pdf]

Reviewer Report

Title: Cell type-specific interpretation of noncoding variants using deep learning-based methods

Version: Original Submission **Date:** 7/4/2022

Reviewer name: Borbala Mifsud

Reviewer Comments to Author:

Gigascience - Cell type-specific interpretation of noncoding variants using deep learning-based methods
Sindeeva et al. have developed DeepCT, a convolutional neural network-based model that predicts sequence and cell type-specific epigenetic profiles from available epigenetic data. The novelty of the approach is that the model can learn unmeasured epigenetic profiles in a given cell type, if there is another cell type that has the target feature measured and shares one or more other epigenetic data types with the cell type it aims to predict in. The authors demonstrated that the framework works well and that the model learns both sequence context and cell type-specificity and used the model to predict which de novo variants, identified in the Simon Simplex Collection, have the highest effect in any of the cell types they studied. Focusing on one variant with high predicted effect in glial cells they suggested a mechanism, whereby the variant in a putative enhancer element within the SMG6 gene reduces FOS binding, which might affect SMG6 expression in these cells. I have a few minor comments to clarify the applicability of this model.

Minor comments:

1. In Figure 2D the authors showed that adult heart and fetal heart cell state representations cluster together even though they did not share the measured epigenetic features. This is an interesting observation, however one of them had ATAC-seq data while the other had DNase-seq data which are highly correlated. It would be good to know how much this can be generalized to other cases. What is the level of correlation between two epigenetic features that is required for correct clustering of the cell states between two cell types that do not share epigenetic features?
2. In both Figure 2C and in Supplementary Figure 1, the 2D visualization of the cell state representations, show that some cell types cluster well together while others do not cluster at all. Even those that cluster well, like "Digestive", "Kidney" or "Muscle" cells have many cell types that do not cluster with the others. Apart from biological differences, could this be also reflective of cell types with lower quality epigenetic tracks? How much does the quality of the tracks effect the model?
3. Figure 3E shows that there are some points where the accuracy of the model is much higher when leaving out certain epigenetic tracks from the training of the model. Is that also related to quality of those data or is there a specific epigenetic feature where the model consistently shows higher accuracy when the feature is left out?
4. The authors used 1000bp for representation of the sequence, but the target sequence that is checked for overlap of the epigenetic features is only 200bp. Does the model learn from the additional 800bp?
5. For the cell state tail the chosen emb_length was 32. Based on Supplementary Figure 1, I assume this is due to the number of cell type groups expected, but it would be good to include the rationale in the methods.

6. For the GO term enrichment what background was used? I would expect that the nearest genes of all de novo variants found in autism cases would show enrichment for similar GO terms.
7. Pg.11 last line should be "FOS transcription factor binding" instead of "grinding".

Methods

Are the methods appropriate to the aims of the study, are they well described, and are necessary controls included? Choose an item.

Conclusions

Are the conclusions adequately supported by the data shown? Choose an item.

Reporting Standards

Does the manuscript adhere to the journal's guidelines on minimum standards of reporting? Choose an item.

Choose an item.

Statistics

Are you able to assess all statistics in the manuscript, including the appropriateness of statistical tests used? Choose an item.

Quality of Written English

Please indicate the quality of language in the manuscript: Choose an item.

Declaration of Competing Interests

Please complete a declaration of competing interests, considering the following questions:

- Have you in the past five years received reimbursements, fees, funding, or salary from an organisation that may in any way gain or lose financially from the publication of this manuscript, either now or in the future?
- Do you hold any stocks or shares in an organisation that may in any way gain or lose financially from the publication of this manuscript, either now or in the future?
- Do you hold or are you currently applying for any patents relating to the content of the manuscript?
- Have you received reimbursements, fees, funding, or salary from an organization that holds or has applied for patents relating to the content of the manuscript?
- Do you have any other financial competing interests?
- Do you have any non-financial competing interests in relation to this paper?

If you can answer no to all of the above, write 'I declare that I have no competing interests' below. If your reply is yes to any, please give details below.

I declare that I have no competing interests.

I agree to the open peer review policy of the journal. I understand that my name will be included on my report to the authors and, if the manuscript is accepted for publication, my named report including any attachments I upload will be posted on the website along with the authors' responses. I agree for my report to be made available under an Open Access Creative Commons CC-BY license (http://creativecommons.org/licenses/by/4.0/). I understand that any comments which I do not wish to be included in my named report can be included as confidential comments to the editors, which will not be published.

Choose an item.

To further support our reviewers, we have joined with Publons, where you can gain additional credit to further highlight your hard work (see: https://publons.com/journal/530/gigascience). On publication of this paper, your review will be automatically added to Publons, you can then choose whether or not to claim your Publons credit. I understand this statement.

Yes Choose an item.
